# Ratiometric Mass Spectrometry Imaging for Stain-Free Delineation of Ischemic Tissue and Spatial Profiling of Ischemia-Related Molecular Signatures

**DOI:** 10.3389/fchem.2021.807868

**Published:** 2021-12-21

**Authors:** Zixuan Wang, Ran Yang, Yaxin Zhang, Xiangyi Hui, Liuyan Yan, Ruiping Zhang, Xin Li, Zeper Abliz

**Affiliations:** ^1^ State Key Laboratory of Bioactive Substance and Function of Natural Medicines, Institute of Materia Medica, Chinese Academy of Medical Sciences and Peking Union Medical College, Beijing, China; ^2^ Center for Imaging and Systems Biology, Minzu University of China, Beijing, China

**Keywords:** mass spectrometry imaging, ischemia, TCA cycle, ratiometric analysis, metabolic enzyme

## Abstract

Mass spectrometry imaging (MSI) serves as an emerging tool for spatial profiling of metabolic dysfunction in ischemic tissue. Prior to MSI data analysis, commonly used staining methods, e.g., triphenyltetrazole chloride (TTC) staining, need to be implemented on the adjacent tissue for delineating lesion area and evaluating infarction, resulting in extra consumption of the tissue sample as well as morphological mismatch. Here, we propose an *in situ* ratiometric MSI method for simultaneous demarcation of lesion border and spatial annotation of metabolic and enzymatic signatures in ischemic tissue on identical tissue sections. In this method, the ion abundance ratio of a reactant pair in the TCA cycle, e.g., fumarate to malate, is extracted pixel-by-pixel from an ambient MSI dataset of ischemic tissue and used as a surrogate indicator for metabolic activity of mitochondria to delineate lesion area as if the tissue has been chemically stained. This method is shown to be precise and robust in identifying lesions in brain tissues and tissue samples from different ischemic models including heart, liver, and kidney. Furthermore, the proposed method allows screening and predicting metabolic and enzymatic alterations which are related to mitochondrial dysfunction. Being capable of concurrent lesion identification, *in situ* metabolomics analysis, and screening of enzymatic alterations, the ratiometric MSI method bears great potential to explore ischemic damages at both metabolic and enzymatic levels in biological research.

## 1 Introduction

Ischemic injury occurs when the blood supply to an organ or tissue is disrupted and causes serious conditions associating with stroke, heart attack, acute kidney injury, and circulatory arrest. After ischemia, both metabolic activities required to maintain the structural integrity and functional activities of the organ are impaired (Hossmann, 1994). Among the metabolism pathways, energy metabolism bears the brunt of disturbance induced by constrained blood and oxygen supply (Belanger et al., 2011). The tricarboxylic acid (TCA) cycle, also known as the citric acid cycle or the Krebs cycle, constitutes an epicenter in cell energy metabolism. It is reported that significant alterations of metabolites in the TCA cycle initiate after ischemic injury (Chan et al., 2018). The most noticeable is accumulation of succinate which controls ischemic-reperfusion injury through mitochondrial reactive oxygen species (ROS) (Chouchani et al., 2014; Andrienko et al., 2017; Chinopoulos, 2019). However, other slight metabolic changes are also worth noticing in exploration of mitochondrial function and ischemia mechanism. Therefore, a method to amplify the slight variations and explore their deep implications is demanded.

Metabolites and metabolic enzymes are important pivots of biological networks whose content and expression directly indicate biological activities of tissue. Spatial characterization of the metabolic or enzymatic alteration in injury tissue may provide region-specific insights into disease-associated mechanism discovery. For example, triphenyl tetrazolium chloride (TTC) staining is currently the most commonly used method for ischemia determination. TTC is used as a redox indicator to differentiate between metabolically active and inactive tissues. When TTC is applied to fresh middle cerebral artery occlusion (MCAO) brain sections, a reaction of TTC and succinate dehydrogenase occurs in living cell mitochondria, turning red in viable tissue by formation of formazan, while the non-viable infarct region remains white (Benedek et al., 2006). Succinate dehydrogenase is one of the key enzymes in the TCA cycle, promoting the transformation of succinate to fumarate and indicating mitochondrial activity in living cells. However, the time-consuming staining procedure and the use of bulk tissue make it difficult to investigate unknown functional features in tissues efficiently.

Mass spectrometry imaging (MSI) has been a prominent technique for profiling of metabolic alterations that enables both high-throughput mapping of molecules and biochemical characterization of tissue morphology. Matrix-assisted laser desorption ionization (MALDI)-MSI and desorption electrospray ionization (DESI)-MSI are efficient tools for MSI analysis of biological samples. MALDI is a highly sensitive soft ionization technique of choice for analysis of macromolecules with quite high spatial resolution. While conventional MALDI needs high-vacuum environments and is influenced by matrix choice, the use of a matrix makes MALDI-MSI of low molecular weight compounds challenging. Neumann et al. introduced trapped ion mobility spectrometry (TIMS) into MALDI-MSI to image low molecular weight metabolites in human kidney, in which the peaks from metabolite signal and matrix can be well separated (Neumann et al., 2020). Spengler and his colleagues designed a MALDI-MSI setup operated at atmospheric pressure using coaxial laser optics (Spengler and Hubert, 2002; Koestler et al., 2008; Guenther et al., 2010), and achieved an ultimate lateral resolution of 1.4 μm for the imaging of subcellular lipids, metabolites, and peptides (Kompauer et al., 2017). DESI-MSI, a represented ambient MSI technique developed by Cooks et al. (Takáts et al., 2004; Wu et al., 2013), can directly extract a wide-coverage of molecular features and visualize their distributions in tissue sections, but the spatial resolution restricts its usage in the elaborate tissues and even single-cell analysis. Our group has developed an ambient MS technology, namely air flow-assisted desorption electrospray ionization mass spectrometry (AFADESI-MS), in which a high-rate air flow is used to facilitate desolvation of secondary droplets and thus improve ion formation during the DESI process. AFADESI-MSI analysis of the metabolome in tissue cryosections, with improved sensitivity and high coverage of metabolite detection (He et al., 2011), has been successfully applied in the whole-body molecular imaging of a large animal sample (Luo et al., 2013), the *in situ* discovery of biomarkers and label-free molecular histopathological diagnosis (Li et al., 2015), and the quantitative analysis of drug distributions in animals (Luo et al., 2018; Song et al., 2019; Wang et al., 2020). MSI has been successfully applied in profiling of cerebral, myocardial, and renal ischemia, and in evaluation of anti-ischemia drugs (van Smaalen et al., 2019; Wu et al., 2019). Liu et al. reported using 1,5-DAN hydrochloride as a matrix for MALDI-MSI of small molecules in focal cerebral ischemia brain tissue, and found alterations in some small molecules after ischemic injury (Liu et al., 2014). Miura et al. used MSI of rat MCAO models to visualize the spatiotemporal behavior of metabolites in the central metabolic pathway (Miura et al., 2010). Hattori et al. combined MSI with capillary electrophoresis and revealed spatiotemporal changes in adenylates and NADH in a mouse MCAO model (Hattori et al., 2010).

In these cases, histological examinations are usually required to annotate specific regions prior to the MSI data analysis and subordinate MSI to the established histopathological results for probing of the region-specific metabolome (Jones et al., 2012). The requirement of sample preparation, staining procedures, and detailed knowledge for manual anatomy interpretation make the pipeline labor-intensive. On the other hand, histology-independent MSI analysis uses molecular histology consisting of MSI datasets to identify which regions have distinct and/or correlated MS profiles. Molecular changes may precede morphometric alterations detectable by histology, potentially providing a more sensitive platform to characterize disease states at an early onset (Woolman et al., 2021). However, histology-independent MSI analysis usually uses the entire MSI dataset to identify specific regions, which may need to develop additional algorithms for reducing dimension and clustering of the huge MSI data (Abdelmoula et al., 2016; Inglese et al., 2019; Guo et al., 2021).

Ratiometic analysis, which uses the signal abundance ratio of two analytes to respond to changes in a specific condition, has been wildly used in mass spectrometry as well as in fluorescence for detection or quantitative measurements of analyte concentrations (Bennett et al., 2008; Zhou et al., 2019; Park et al., 2020). For example, in absolute quantitative proteomics using stable isotope labeling, samples to be analyzed for quantification are differentially labeled with a stable isotope, combined, and simultaneously subjected to MS. The ratio of peak intensity between the ions of an isotope pair (i.e., light and heavy ion species) gives relative difference in abundance of the compound (Thompson et al., 2003; Kito and Ito, 2008; Yuan et al., 2018). Bennett et al. reported a protocol for quantitating the intracellular concentrations of endogenous metabolites in cultured cells grown in stable isotope-labeled media. The ratio of endogenous metabolite to internal standard in the extract is determined using mass spectrometry (Bennett et al., 2008). Zhao et al. used non-isotopic mass tags for ratiometric quantitation to eliminate variations in ionization efficiencies due to heterogenous sample matrixes (Zhao et al., 2018). These studies have shown the great possibility of the ratiometric method in MS analysis to eliminate intrinsic and systematic errors. In 2019, our group reported a spatially resolved metabolomics method for the discovery of tumor-associated metabolite and enzyme alterations based on AFADESI-MSI (Sun et al., 2019), in which ratiometric analysis was performed to find region-specific enzymes upon the direction of histopathological information.

**FIGURE 1:**
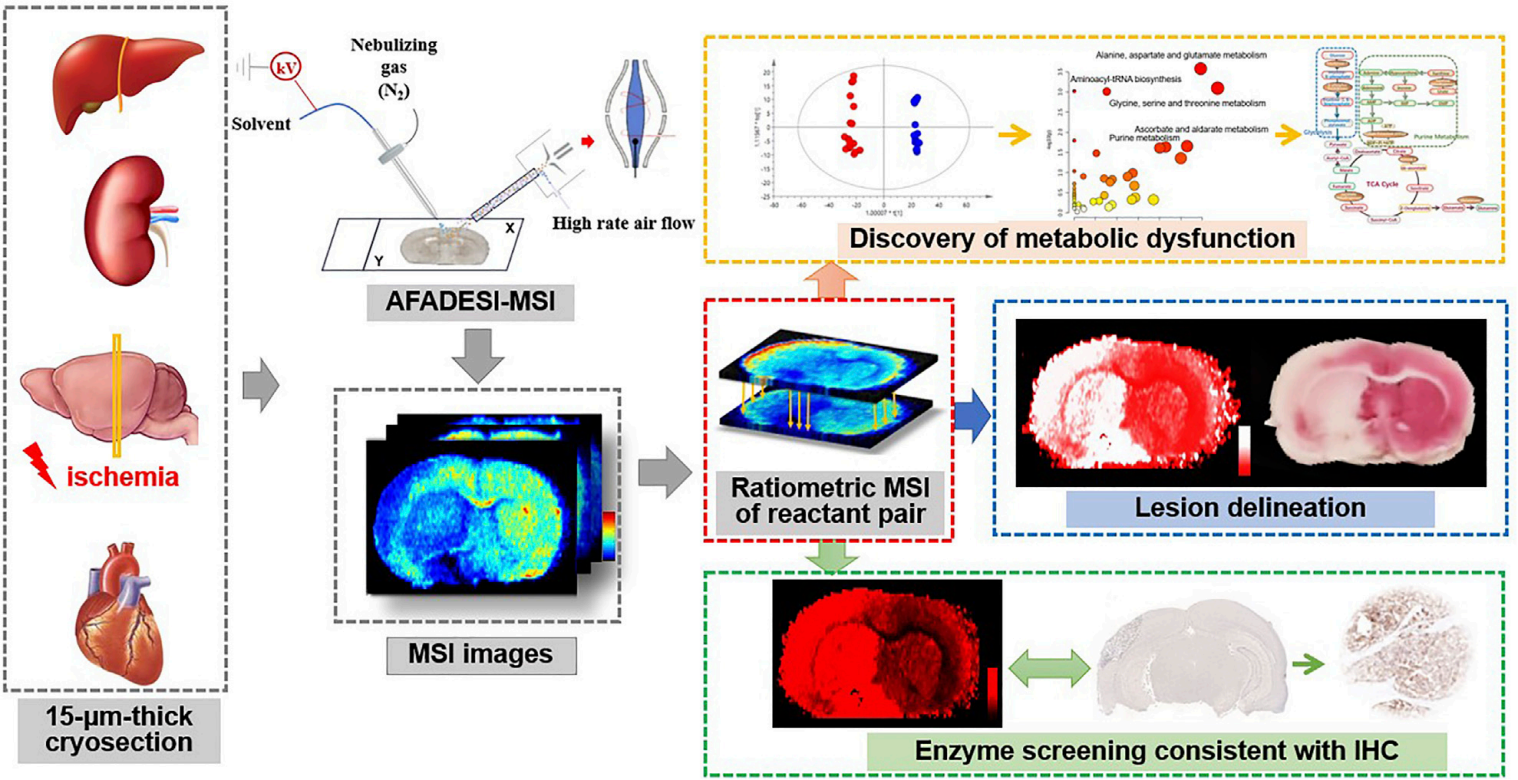
Schematic illustration of the ratiometric mass spectrometry imaging strategy for delineation of ischemia and spatial profiling of metabolic and enzymatic signatures.

The metabolic reactant pairs, namely substrate and product of the reaction, are usually similar in tissue distribution and chemical structure. Therefore, *in situ* measuring of the ratio of the reaction pair is thought to help eliminate systematic error and reduce the variations of MS signal of metabolite ions in heterogeneous tissue. In this study, we propose an *in situ* ratiometric MSI strategy for stain-free delineation of the lesion area and discovery of ischemic-associated metabolic and enzymatic alterations. Ischemia-induced metabolic alterations in the TCA cycle are firstly profiled in detail by AFADESI-MSI. Then, we define the pixel-by-pixel ion abundance ratio of the reactant pair in the tissue cryosection as an indicator for a spatially resolved receiver operator characteristic curve (ROC) analysis. The method is applied for discrimination of an ischemic lesion and the discovery of metabolic and enzymatic alterations. The applicability of the method is tested in a variety of animal ischemic models. The design of this strategy is shown in Figure 1.

## 2 Materials and Methods

### 2.1 Solvents and Reagents

LC-MS grade acetonitrile (ACN) was purchased from Fisher Scientific Co., Ltd. (Fair Lawn, NJ, United States). Purified water was obtained from Wahaha (Hangzhou, China). Reference standards of metabolites in the TCA cycle were purchased from Sigma Aldrich (Merck KGaA, Darmstadt, Germany). Anti-aconitase 2 antibody (ab 129069) was obtained from Abcam (Cambridge, United Kingdom).

### 2.2 Animal Studies

All animal experimental procedures were in accordance with the standards set forth in the guide for the care and use of laboratory animals approved by the Animal Care and Welfare Committee, Institute of Materia Medica, Chinese Academy of Medical Sciences and Peking Union Medical College (Beijing, China). Animals including Wistar and Sprague-Dawley (SD) rats and Kunming mice were purchased from Vital River Laboratory Animal Technology Co. [SCXK(Jing) 2016-0006, Beijing, China]. Animals were housed under standard temperature and humidity, diurnal lighting conditions, and allowed food and tap water ad libitum.

#### 2.2.1 *In Vivo* Rat Brain Ischemia and Reperfusion

Focal cerebral ischemia was established by permanent and transient middle cerebral artery occlusion (MCAO) on male Wistar and SD rats (300–350 g). The rats were anesthetized by isoflurane inhalation. The right common carotid artery (CCA), external carotid artery (ECA), and internal carotid artery (ICA) were isolated. Then, a standardized nylon suture was inserted from the ECA into the ICA until the marker on the suture was shown on the intersection of ECA and ICA, which indicated that the suture successfully blocked the middle cerebral artery (MCA). For transient MCAO (tMCAO) models, the nylon suture was removed to start reperfusion after 90 min and re-perfused for 3, 6, 12, and 24 h. The permanent MCAO (pMCAO) models were divided into 1-day and 7-days groups. The rats were evaluated with modified nerve function defect (mNSS) score to ensure whether the model was successfully built.

#### 2.2.2 *In Vivo* Rat Myocardial Ischemia and Reperfusion

Male SD rats (300–350 g) were anesthetized by isoflurane inhalation. A thoracotomy was performed and the heart was exposed by stripping the pericardium (Methner et al., 2013). A prominent branch of the left anterior descending coronary artery was surrounded by a prolene suture that was then passed through a small plastic tube. Ischemia was induced by tightening the tubing against the heart surface (Chouchani et al., 2014). The tubing was removed after 60 min of ischemia for reperfusion.

#### 2.2.3 *In Vivo* Mouse Renal Ischemia and Reperfusion

Kunming mice (30–36 g) were anesthetized by isoflurane inhalation. Mice underwent laparotomy and exposure of the renal hilum bilaterally. Vascular clips were placed over one renal hila to induce unilateral renal ischemia. At the end of 60 min of ischemia, the clip was removed and reperfusion of the kidney was noted as a return of blush color and visualization of flow from the renal vein (Chouchani et al., 2014).

#### 2.2.4 *In Vivo* Rat Liver Warm Ischemia

Rats were sacrificed by decapitation after anesthesia to ensure cessation of blood flow. Liver tissue was maintained *in situ* in the body cavity for 60 min at 37°C through use of a thermostated heat pad (Chouchani et al., 2014).

### 2.3 Tissue Sample Preparation

Target tissues were acquired after sacrificing animals by decapitation with anesthesia. Tissues were stored at −80°C until sectioned at 15-μm thickness using a CM 1860 UV cryostat microtome (Leica, Nussloch, Germany). The tissue sections were thaw-mounted onto a microscope slide and stored in closed containers at −80°C. Before AFADESI-MSI analysis, the microscope slides were dried in a vacuum for 60 min.

### 2.4 AFADESI-MSI Analysis

The AFADESI-MSI platform was built by replacing the original ion source of a Q-Orbitrap mass spectrometer (Q Exactive, Thermo Scientific Bremen, Germany) with a home-built AFADESI ion source. A high-rate air flow was used to facilitate ion formation and collection. A 500-mm-long stainless tube (OD 4 mm, ID 3 mm) was installed to transform ions. An XY translational stage controlled by custom-developed software placed under the ion source and SC100 series stepper motor (Beijing Optical Century Instrument Co., Beijing, China) was used to control the platform.

AFADESI-MSI analysis was performed in positive- and negative-ion mode on a Q-Exactive mass spectrometer at the mass range of *m/z* 70-1000. Tissue sections were placed on the 3D translational stage with a moving step size of 0.2 mm/s horizontally and 0.2 mm vertically. A mixed solution of acetonitrile and water (8:2, vol/vol) was used as the spray solvent with a flow rate of 5 μL/min. The spatial resolution of the AFADESI-MSI setup in this experiment was around 100 μm. The high-rate air flow was set to 40 L/min and the capillary temperature was 350°C.

### 2.5 Histological Examinations

#### 2.5.1 TTC Staining

Two millimeter brain slices adjacent to the sections analyzed by AFADESI-MSI were immersed in 2% TTC solution and incubated at 37°C for 30 min in the dark. The ischemic area was stained white, and the non-ischemic area turned red.

#### 2.5.2 HE Staining

Tissue sections were stained with hematoxylin and eosin (HE) staining solution. Images were visualized by a Panoramic MIDI scanner (3DHISTECH, Budapest, Hungary) and analyzed by CaseViewer (3DHISTECH Ltd., Hungary).

#### 2.5.3 Immunohistochemistry

Expression of aconitase 2 in the rat brain tissue sections which were homologous with the ones analyzed by AFADESI-MSI were assessed. The frozen tissue sections were first fixed in 4% paraformaldehyde for 10 min. Then, the sections were immersed in 0.25% Triton X-100 for 15 min and blocked with 1% bovine serum albumin for 30 min. After being incubated with targeted antibodies (1:200) at 4°C overnight, images were visualized by a Panoramic MIDI scanner (3DHISTECH, Budapest, Hungary) and analyzed by CaseViewer (3DHISTECH Ltd., Hungary).

### 2.6 Data Processing

The collected raw data from AFADESI-MSI were converted to. cdf files by Xcalibur software. Then custom-developed imaging software MassImager Pro (a dedicated imaging software based on the C++ programming language) was used for ion image reconstructions and pattern recognition multivariate statistical analysis. The pixel-by-pixel ion abundance ratio image of the reactive pair and segmentation of the lesion were constructed by MATLAB 2018a (The MathWorks, United States) with a self-defined code (Supplementary Material).

### 2.7 Statistical Analysis

Next, 5*5-pixel ROIs were selected and their average mass spectra were rearranged and normalized into a 2D matrix with Makerview software 1.2.1 (AB Sciex, Framingham, Massachusetts, United States). Further multivariate statistical analysis was done using SIMCA version 14.0.1 (Umetrics AB, Umea, Sweden). Student t-test and Mann-Whitney U test were performed with SPSS (Statistical Product and Service Solutions v.24.0, IBM Corp., NY, United States).

### 2.8 Metabolite Identification

The ion features of interest were first compared with on-line database HMDB (http://hmdb.ca/), using exact molecular weights and a mass accuracy of less than 5 ppm, combining the isotope abundance from HRMS to give the elemental composition and possible list of endogenous metabolites. Further, *in situ* MS/MS analysis of the representative metabolites was performed directly on tissue sections with a resolution of 17,500. Representative metabolites were also verified by a high performance liquid chromatography system (SHIMADZU LC 20, Tokyo, Japan) coupled to a TripleTOF 5600 + mass spectrometer (AB Sciex, Concord, ON, Canada) and compared with reference standards. The fragment ions were compared and identified through MetFrag (https://msbi.ipb-halle.de/MetFrag/).

## 3 Results and Discussion

### 3.1 Spatial Mapping of Energy Metabolic Dysfunction After Ischemia Using Ambient MSI

**FIGURE 2:**
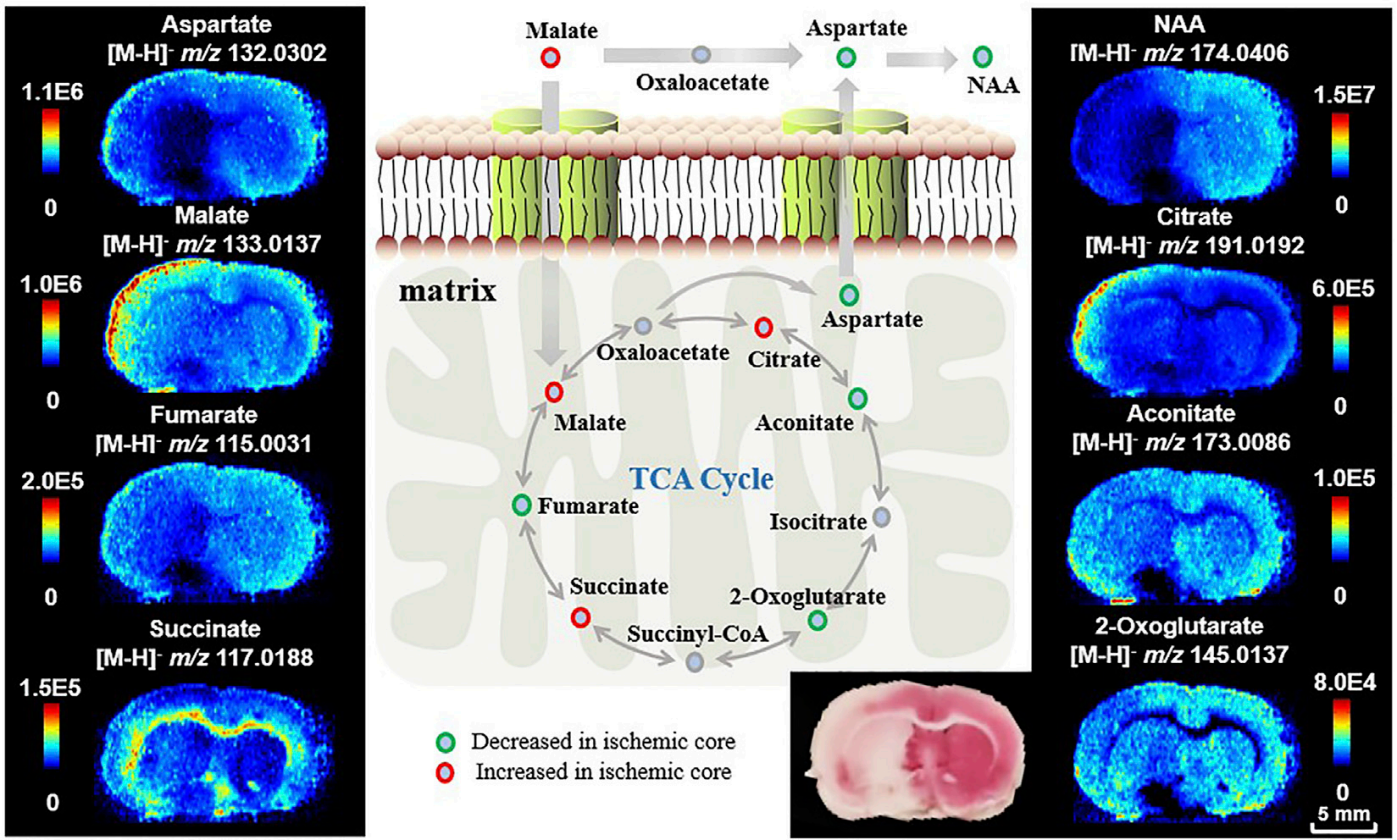
Ion images of metabolites and pathways related to energy metabolism after ischemia. The ipsilateral cerebral hemisphere underwent focal ischemic/reperfusion injury by tMCAO.

Energy metabolism bears the brunt of disturbance induced by ischemia. Providing major energy resources in metabolism, the TCA cycle is important for biosynthesis of molecules and thus determines the fate of tissue viability (Belanger et al., 2011). Here, we firstly target metabolites in the TCA cycle in cryosections of 24-h MCAO rat brains, guided by the TTC staining result of the adjacent sections to find spatial differences between ischemic injury and healthy tissue. Figure 2 shows AFADESI-MSI analysis of TCA cycle metabolites in a coronal section of MCAO rat brain. Compared to the healthy cerebral hemisphere, fumarate (*m/z* 115.0031, [M-H]^–^), aconitate (*m/z* 173.0086, [M-H]^–^), and 2-oxoglutarate (*m/z* 145.0137, [M-H]^–^) are significantly downregulated in the ischemic core, while malate (*m/z* 133.0137, [M-H]^–^) and citrate (*m/z* 191.0192, [M-H]^–^) are markedly increased in the injured area, especially in the cortex of the ischemic hemisphere. Succinate (*m/z* 117.0188, [M-H]^–^) is accumulated in the inner layer of the cortex and white matter in the ischemic region, which may be related to its structural distribution and role in ROS production (Andrienko et al., 2017; Chinopoulos, 2019). In addition, the malate-aspartate shuttle (MAS) constitutes the primary metabolic pathway for transfer of reducing equivalents from cytosol into the mitochondria for oxidation. We also find a decrease in aspartate (*m/z* 132.0302, [M-H]^–^) and its downstream metabolite N-acetyl aspartate (NAA, *m/z* 174.0406, [M-H]^–^), the latter is recognized as a biomarker for neuron integrity (Moffett et al., 2007). Identification of metabolites using MS/MS analysis and statistical results of the related metabolites are shown in Supplementary Table S1 and Supplementary Figures S1–S8. Compared to previous studies (Liu et al., 2014; Wu et al., 2019), AFADESI-MSI gives a comprehensive profiling of metabolites in the TCA cycle. These results suggest significant alterations of energy-related metabolites and pathways and loss of mitochondrial function after ischemia.

### 3.2 Ratiometric MSI for Delineation of Ischemia in Biotissues: Concept and Principle

Delineation of lesion regions using a single biomarker may be interfered by its complex structural and functional heterogeneity. Besides, the chemical noise arising from tissue biomatrixes makes direct mapping of the lesion region using a small number of metabolic biomarkers challenging. Ratiometric quantitation (ratio of increasing signal over decreasing signal) is a preferred method in spectroscopy as it corrects for artificial changes. It has also been used in mass spectrometry to account for variations in ionization efficiencies due to heterogenous sample matrixes (Cocheme et al., 2011; Cocheme et al., 2012; Zhao et al., 2018). Considering that the content and distribution of metabolites are closely related to metabolic conversions, we propose an *in situ* ratiometric MSI method using the ion abundance ratio of the reactant pair (substrate-product pair) to amplify metabolic alteration during ischemic injury and characterize tissue viability. ROC curve analysis is well established for assessing how well the indicator is capable of discriminating between individuals who experienced disease onset and those who did not. Here, we define the ion abundance ratio of the reactant pair as an indicator for diagnosis of ischemic injury. Particularly, we perform a spatially resolved diagnosis using AFADESI-MSI with 100 × 100-μm spatial resolution. That is to say, ratiometric MSI is reconstructed by assessing the ion abundance ratio of the reactant pair in each pixel, and the cut-off value of ROC curve analysis is used for discriminating the ischemic and healthy pixels and their boundary. Therefore, compared to the TTC staining which depends on coarse recognition of injury regions, the improvement of the ROC curve in ratiometric MSI gives a precise delineation result with 100-μm resolution. It can be prospected that this *in situ* ratiometric analysis method can be implemented on the high spatial resolution MSI system, i.e., laser desorption-based approaches, to precisely explore the molecular signatures at the single-cell level.

**FIGURE 3:**
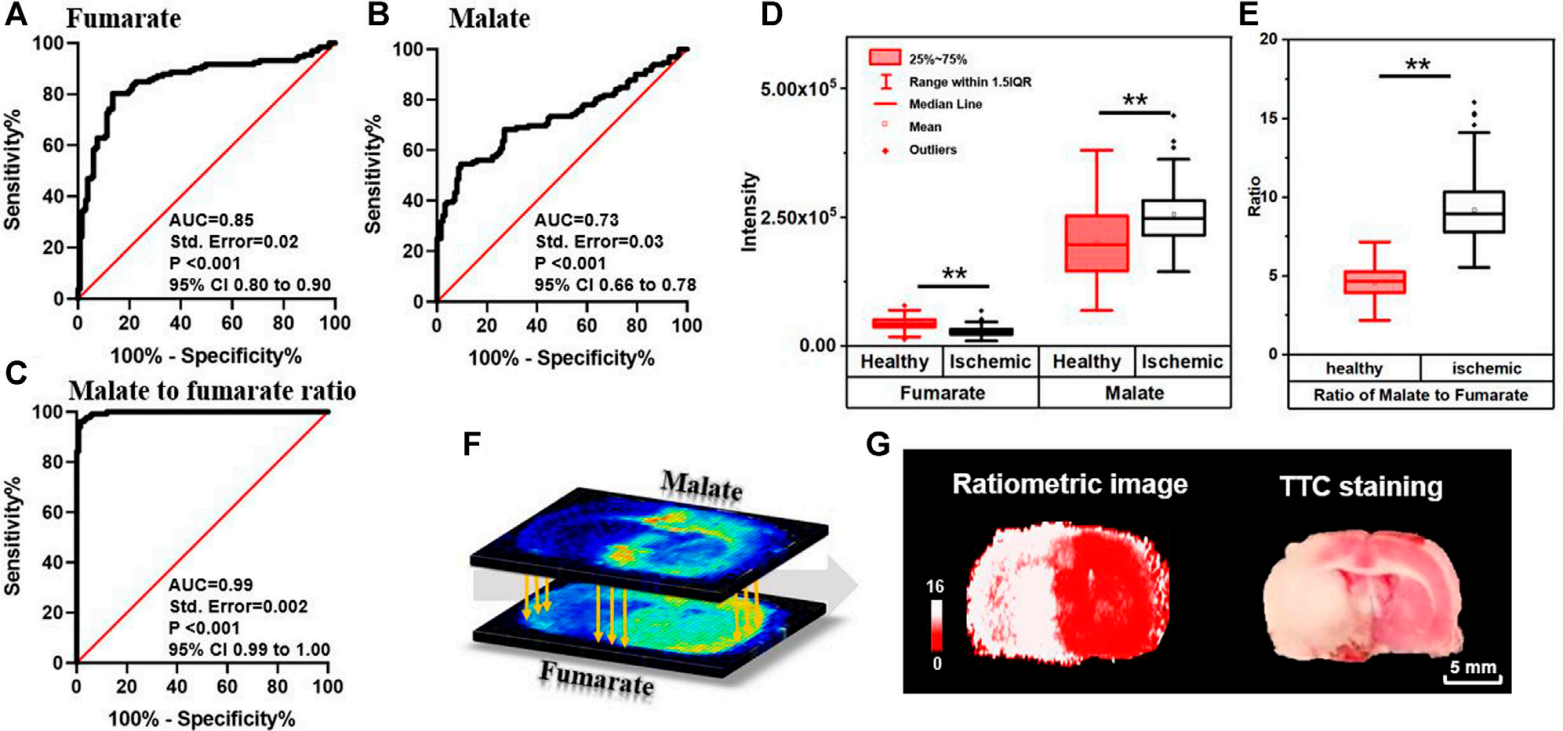
ROC curves of fumarate **(A)**, malate **(B)**, and ratio of the malate to fumarate reactant pair **(C)** for diagnosing ischemia; box plots of malate, fumarate **(D)**, and their ratio **(E)** in ischemic and healthy regions in MCAO rat brain sections (*n* = 265, **p < 0.01); **(F)** schematic diagram of pixel-by-pixel ratiometric MSI of malate to fumarate as a reactant pair for ischemic delineation; **(G)** the reconstructive ratiometric image and the image of adjacent section after TTC staining.

As shown in Figure 2, tissues in the ischemic core of focal infarction are devitalized and the metabolites in the TCA cycle are significantly altered. Malate and fumarate are two metabolites mutually transformed by a one-step reaction in the TCA cycle. The distribution of neither malate nor fumarate can be used directly for discrimination of ischemia compared with the result of TTC staining, whereas the inversely correlated alterations of malate and fumarate (Figure 3D) indicate a vulnerability of ischemic injury. Figures 3A,B illustrate ROC curves of fumarate and malate, respectively, for diagnosing ischemic regions from 265 ROIs of different MCAO models selected from ischemic and corresponding healthy hemispheres with the guide of adjacent TTC staining, with AUCs of 0.85 and 0.73, respectively. This indicates that these two metabolites have the ability to identify ischemia, yet the diagnosability needs to be improved. When using the defined ion abundance ratiometry of malate to fumarate for diagnosis, the diagnosability is well improved. As shown in Figure 3C, the AUC of the ROC curve increases to 0.99, which helps for enhancement of both diagnosis sensitivity and specificity. The cut-off value of the ratiometric ROC curve is 6.2596, and the fold change is improved from 1.2 to 2-fold (Figures 3D,E). Supplementary Figure S9 shows the extracted ion chronograms (EIC) of malate, fumarate, and their ratio in a 1.6-min AFADESI-MSI scan of a MCAO section. Compared to using a single metabolite, ratiometry offers a remarkable transition value for profiling the ischemic zone.

The method is further developed for spatial profiling and locating ischemia regions by firstly reconstructing pixel-by-pixel ion abundance ratio (Figure 3F) and then performing classification with the calculated cut-off value. This process is performed by using a self-defined MATLAB code (shown in Supplementary Material). As illustrated in Figures 3E,G, pixels with a ratio higher than 6.2596 are shown in white for ischemia and the ones lower than 6.2596 are shown in red for vitality tissue, which precisely profiles ischemic boundary and is consistent with the result of the adjacent MSI tissue section for TTC staining. The proposed method bears the advantage of a stain-free delineating injury area by magnifying metabolite alterations and neglecting structural differences. Furthermore, resembling the absolute quantitation with stable isotope labeling, the *in situ* ratiometric analysis eliminates instrumental error and errors from system instability by determining the pixel-by-pixel ratio within the same acquisition. In this ratiometric MSI method, quantitative values were calculated based on the ratio of peak intensity between reactant pair ions. By sampling from the same spot and having similar chemical structures, ions of the reactant pair can be simultaneously introduced into the mass spectrometer but clearly distinguished by their mass. Simultaneous measurement of ion intensities in the same scan alleviates not only run-to-run variations in the performance of MS, region heterogeneity, and ion-suppression effect of co-desorbing ions, but also limits the intrinsic dynamic range of each MS, thereby enabling more accurate and reliable quantification. The results demonstrate that the ratiometric MSI method has the potential to emerge as a complementary technique to the absolute quantitation for MSI.

### 3.3 Delineation of Lesion and Spatiotemporal Profiling of Metabolic Signatures in Ischemic Tissue

**FIGURE 4:**
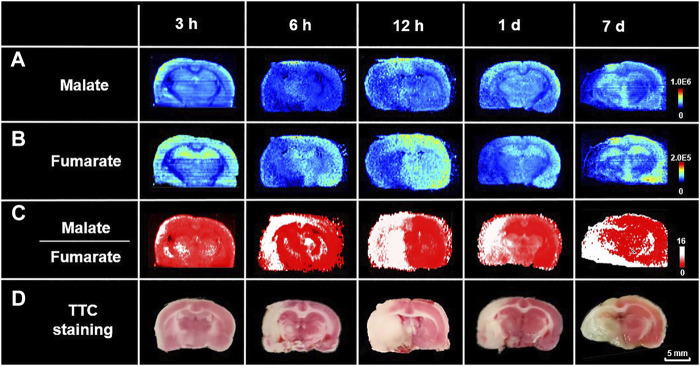
Spatiotemporal profiling of metabolic signatures and delineation of ischemia using ratiometric MSI. Ion images of **(A)** malate, **(B)** fumarate, and **(C)** reconstructed ratiometric images of malate to fumarate, respectively, by AFADESI-MSI analysis of rat MCAO brain sections with different ischemic durations. **(D)** Images of TTC staining of their adjacent sections.

To test the robustness of the suggested strategy, we made MCAO models of different ischemic durations as samples for delineation of lesion and spatiotemporal profiling of metabolic signatures. Figures 4A,B show the ion images of malate and fumarate, respectively, on the MCAO rat brain sections with 3, 6, 12-h and 1, 7-days ischemia. Samples with different ischemic durations show an upregulated trend of malate and downregulated trend of fumarate in the ischemic hemisphere. Ratiometric images are reconstructed with the pixel-by-pixel ion abundance ratio of malate to fumarate and classified by the above cut-off value from ratiometric ROC curves (Figure 3C). As shown by the reconstructive ratiometric images in Figure 4C, the neocortex of a 3-h MCAO rat brain was subjected to ischemic injury. However, this cannot be identified by TTC staining in which early determination (4 h or less) is equivocal or inaccurate (Hatfield et al., 1991; Benedek et al., 2006). Therefore, the ratiometric MSI approach has the advantage of more sensitive ischemic discrimination than the traditional staining approaches. After 6 h of ischemia, edema occurred and the cortex was injured by infarction. While after 12–24 h of ischemia, the volume of infarction was enlarged and the ischemic injury expanded to the structures like striatum, thalamus, and globus pallidus. In the 7-days model, the ipsilateral side atrophied in volume and colliquative necrosis occurred in the cortex, thalamus, and CA1 region in the hippocampus. All the rest results meet with that of TTC staining with adjacent sections of MSI (Figure 4D). The proposed method demonstrates the significant potential of ratiometric MSI to spatially map ischemic signatures accurately in brain injuries, with high sensitivity, good robustness, and independent of traditional histology-guided approaches.

More importantly, the method also bears the capability of concurrent lesion identification and *in situ* metabolomics analysis within a 15-μm-thick cryosection, circumventing tissue consumption, tedious operation for staining, and artificial errors. Supplementary Figure S10 gives the ischemic metabolome analysis results after lesion identification by the proposed method. Mann-Whitney U test combined with orthogonal partial least squares discriminant analysis (OPLS-DA) analysis enabled discovery of altered metabolites after infarction. As shown in Supplementary Figure S11, a total of 565 ion features are found significantly changed after ischemia, with annotated metabolite types of lipids, organic acids, amino acids, nucleosides and nucleotides, carbohydrates, and carnitines, etc. These results represent the wide coverage and high throughput of AFADESI-MSI analysis. The discriminating metabolites are further imported into the Kyoto Encyclopedia of Genes and Genomes (www.kegg.jp) to perform metabolic pathway matching analysis, facilitating the discovery of altered metabolic pathways. The results are presented in Supplementary Figure S12, where arginine and proline metabolism, glycine, serine, and threonine metabolism, ascorbate and aldarate metabolism, and purine metabolism are found significantly dysregulated after ischemic injury.

### 3.4 Applicability of Ischemic Delineation Using Ratiometric MSI

**FIGURE 5:**
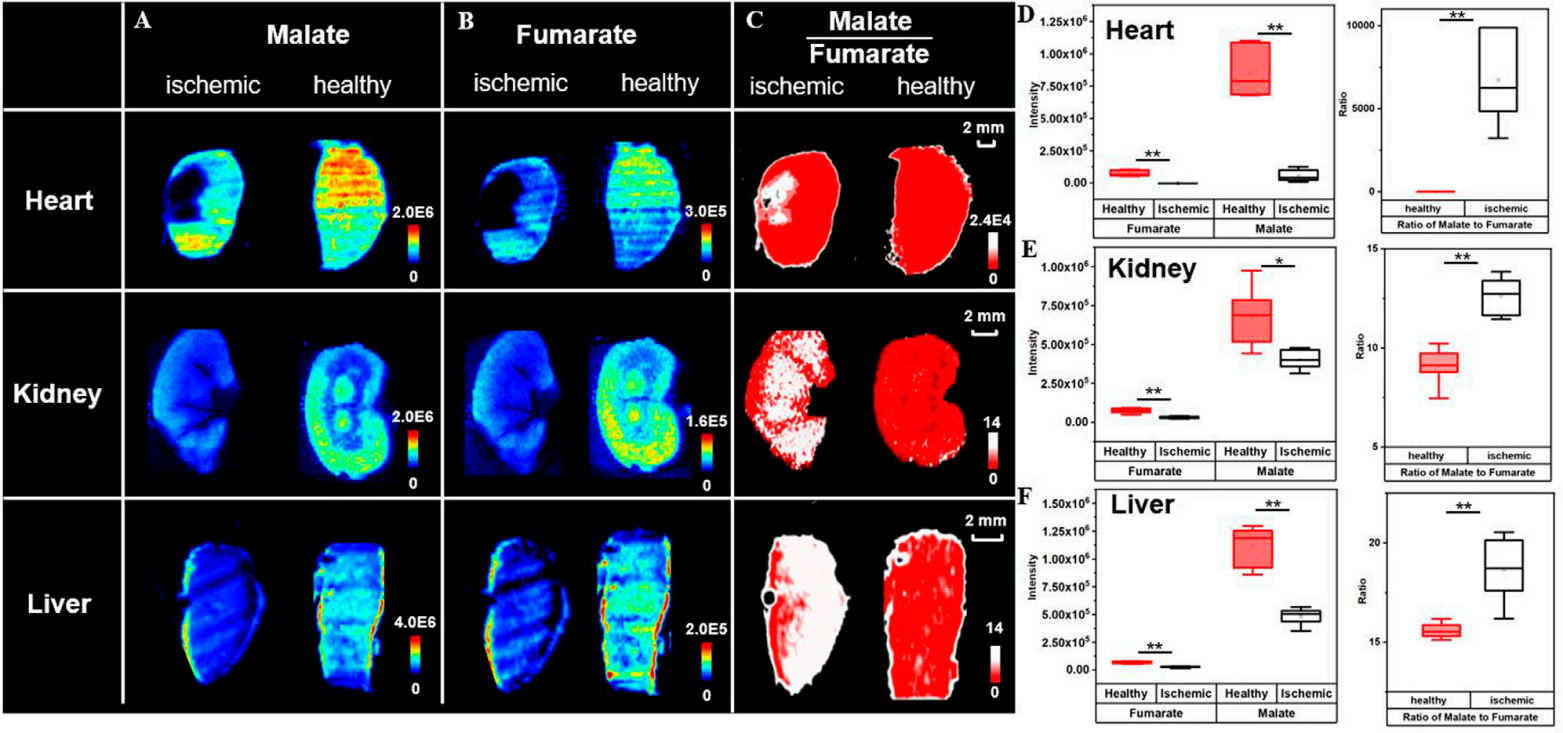
Broad application of ischemic delineation using ratiometric MSI. Ion images of malate, fumarate, and their ratio for delineation of ischemia **(A–C)**. Box plots represent their abundances **(D–F)** in different organs. (*n* = 6, *p < 0.05, **p < 0.01).

To further test the applicability of the proposed strategy, the rat myocardial ischemia and reperfusion models, the mouse renal ischemia and reperfusion models, and the rat liver warm ischemia models are built. Their cryosections are prepared for ratiometric MSI to distinguish lesion from healthy tissue regions. Figure 5A shows ion images of malate and fumarate in the rat myocardial ischemia and reperfusion group, and the sham group. Different from that in MCAO rat brain, the necrotic area in the heart shows a decrease in abundances of both malate and fumarate compared to the healthy region and the sham group. The 14-fold decrease of malate and nearly disappearance of fumarate roughly give a 6500-fold increase in the abundance ratio of ischemic area in the myocardium (Figure 5D). In the mouse renal ischemia and reperfusion models and the pairing sham groups, the ratio of malate to fumarate is also higher than that in the sham group (Figures 5B,E). The liver warm ischemia models give a similar result with a higher ratio value in the model group compared with the normal liver (Figures 5C,F).

Regardless of cut-off values for different models and trends in metabolite alteration, the high ratio of the malate to fumarate reactant pair can universally distinguish ischemic lesions (regions in white) from healthy tissues (regions in red). The discriminated lesions are later confirmed through the microscope observation of HE staining results of adjacent MSI sections. Compared with the normal tissue, the tissue delineated as ischemia is looser in cell arrangement and shows intracellular damage of nuclear pyknosis and signs of vacuolization (Supplementary Figure S13). Compared to the use of NAA, a well-known biomarker for neuronal integrity, which does not show variation in ischemic liver (Supplementary Figure S14), the ratiometric MSI approach gives a universal and robust insight into ischemic injury delineation. The increase of malate to fumarate ratio in kidney ischemia has also been proved by a study of Nielsen et al. using magnetic resonance spectroscopy (Nielsen et al., 2017). The results above demonstrate the broad applicability of the ratiometric MSI of the reactant pair, malate to fumarate, to spatially delineate ischemic area in different organs, models, and with different ischemic durations.

### 3.5 Screening and Prediction of Enzyme Expression Using Ratiometric MSI

**FIGURE 6:**
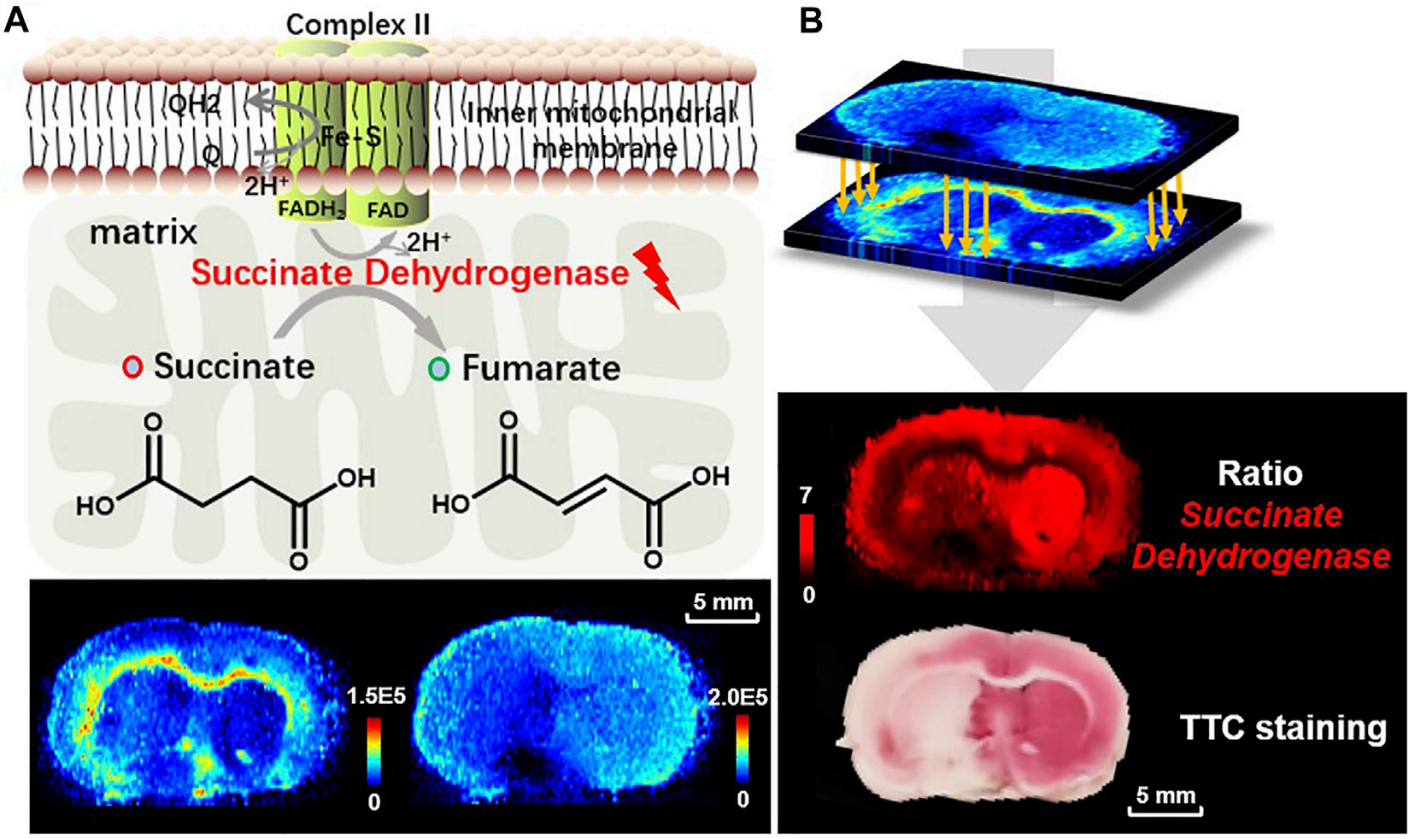
**(A)** The reaction of succinate to fumarate catalyzed by SDH in mitochondria, and the ion images of fumarate (*m/z* 115.0031, [M-H]^−^) and succinate (*m/z* 117.0188, [M-H]^−^) in the MCAO brain tissue; **(B)** reconstructed ion image of the abundance ratio of the reactant pair, fumarate to succinate, and TTC staining for validation of SDH reduced expression.

All kinds of diseases, including ischemia will cause *in vivo* metabolic disorder. As the node of metabolic pathways, metabolic enzymes play a key role in metabolite interactions and tissue vitality. Metabolites are the direct products or substrates of metabolic enzymes, and the level of metabolites in tissue may reflect enzyme capacities (Fendt et al., 2010; Sun et al., 2019). In a metabolic pathway, a reactant pair with inverse-correlated alteration trends may indicate a metabolic vulnerability and is thus a possible therapeutic target. Therefore, we further broaden the application of the proposed ratiometric MSI method to predict the catalyzing enzyme expression of the specific reactant pair. A case in point is the prediction of succinate dehydrogenase (SDH), which is found in the inner mitochondrial membrane and provides electrons for the aerobic respiratory chain (known as complex II), catalyzing the reciprocal reaction of succinate to fumarate in the TCA cycle (Figure 6A). As illustrated in Figures 2, 6A, fumarate is downregulated while succinate is upregulated after ischemia. Here we use the ion abundance ratio of succinate to fumarate to reconstruct the expression of succinate dehydrogenase. As shown in Figure 6B, the reconstructed image indicates a decrease in enzyme expression in ischemic areas such as the thalamus and the cortex of the ipsilateral side. The delineation of enzymatic boundary is verified by TTC staining with good consistency, in which the SDH deficiency tissue is shown in white.

**FIGURE 7:**
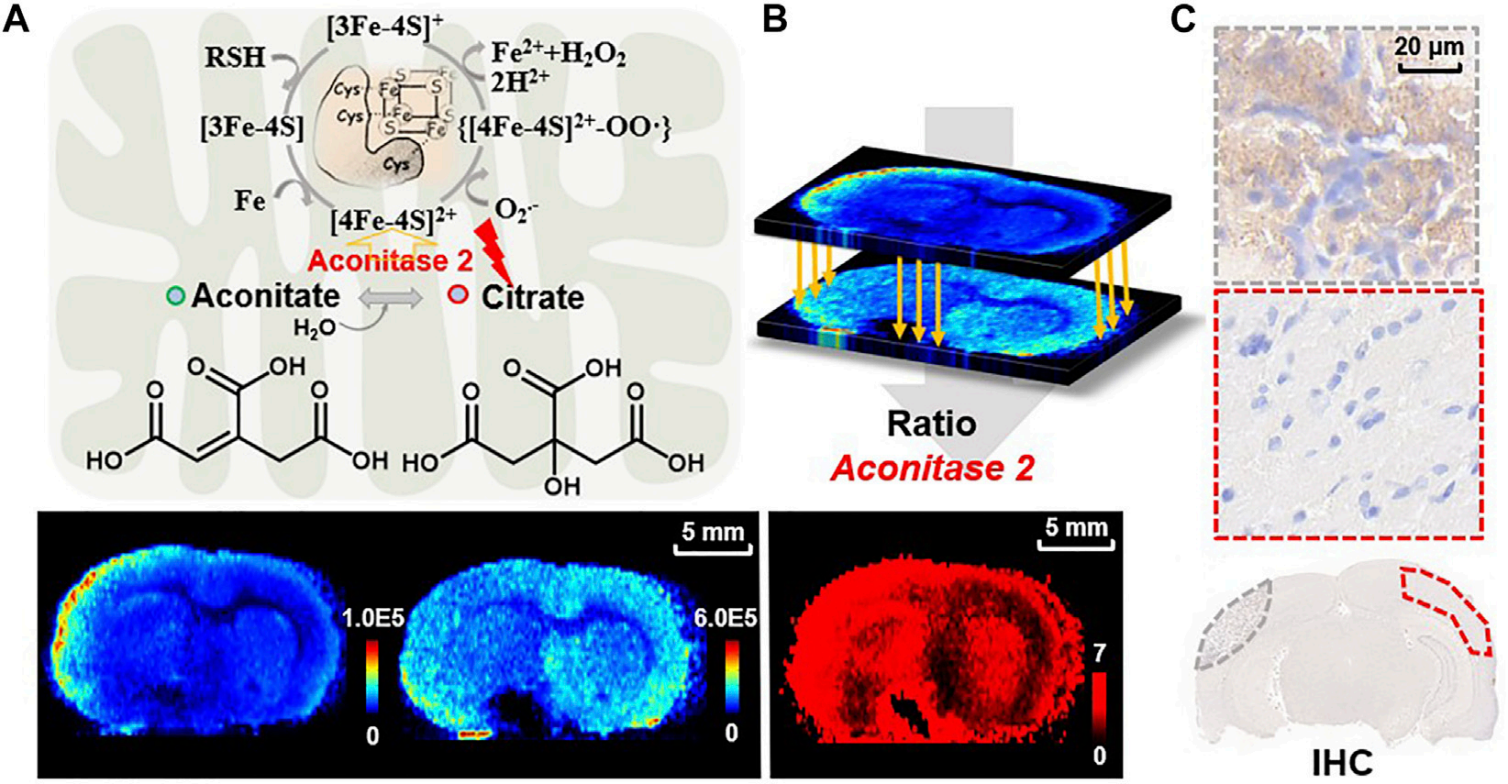
**(A)** The reaction of aconitate to citrate catalyzed by aconitase 2 in mitochondria and the related mechanism of superoxide-mediated generation of hydroxyl radical from aconitase 2 prosthetic group. Ion images of aconitate (*m/z* 173.0086, [M-H]^−^) and citrate (*m/z* 191.0193, [M-H]^–^) in the MCAO brain tissue; **(B)** reconstructed ratiometric image of the reactant pair of citrate to aconitate and **(C)** IHC for validation of aconitase 2 expression in MCAO rat brain sections (dotted lines representing the sampling zone).

To further test the capability of ratiometric MSI of the reactant pair for predicting enzymatic alteration, we focus on the three reactant pairs with inverse-correlated alteration in the TCA cycle as shown in Figure 2. Apart from the reactant pairs of fumarate to succinate for decrease of SDH and malate to fumarate for increase of fumarate hydratase (Nielsen et al., 2017) mentioned above, alterations in abundance of citrate and aconitate are also worth noting. This reaction is catalyzed by aconitase 2, which represents a key metabolic hub for satisfying bioenergetic and biosynthetic requirements. Aconitase 2 has also been considered to be a source of ROS formation (Vasquez-Vivar et al., 2000), for it contains a (4Fe-4S) prosthetic group in the active center, which is susceptible to ROS. Oxidation of the [4Fe-4S]^2+^ cluster of aconitase 2 by O_2_^•—^ results in the release of Fe^2+^ and H_2_O_2_ from aconitase 2 and produces highly cytotoxic •OH *via* a reaction between Fe^2+^ and H_2_O_2_, which is commonly referred to as the Fenton reaction (Nakajima et al., 2015) (Figure 7A). Aconitase 2 has been proved to be harmful to neurons when exposed to oxidative stress and its overexpression resulted in a further increase in H_2_O_2_ production and neuronal cell death (Cantu et al., 2009). As shown in Figure 7A, aconitate is slightly downregulated in the ischemic area while citrate is upregulated in the cortex of the lesion area. Again, we use the pixel-by-pixel ion abundance ratio of citrate to aconitate to delineate aconitase 2 expression. The result shows aconitase 2 is overexpressed in the ipsilateral cortex compared to the contralateral side (Figure 7B). In Figure 7C, IHC testing of the homologous tissue verified our discovery that the expression of aconitase 2 is higher in the ischemic cortex (dotted line in gray) than that of the contralateral side (dotted line in red) on the same brain section. A previous proteomic analysis is in accordance with our result showing an increase in expression level of aconitase 2 in the cerebral cortex after infarction (Chen et al., 2014). This is possibly due to the *in situ* production of ROS induced by mitochondrial ischemia-reperfusion injury which affects aconitase 2, leading to the exacerbation of oxidative stress and the overexpression of aconitase 2. The MSI ratiometric method of the reactant pair provides potential for high throughput screening of the dysregulated enzymes and sheds light on the metabolic-based therapy by targeted inhibition or inducement of the dysregulated enzymes or altering the levels of downstream metabolites.

## 4 Conclusion

In summary, a ratiometric MSI method has been established for convenient identification of ischemic lesions, *in situ* metabolomics analysis of tissue ischemia, and thereby highly efficient screening of enzymatic alterations in energy metabolism on identical tissue sections. In this approach, the inversely correlated ion abundance ratio of the reactant pair is utilized to amplify the metabolic alterations in the ischemic tissue and serves as a damage localization indicator for discrimination of ischemic zone by taking advantage of circumventing morphological mismatch and tissue consumption. The feasibility of the ratiometric MSI approach has been validated in different ischemic models including brain, heart, liver, and kidney with different ischemic durations, demonstrating its robust ability to identify lesions. The TTC and IHC staining results have suggested that the metabolic reactant pairs with inversely correlated abundances can be used for predicting enzymatic alterations. We have assessed three altered metabolic enzymes targeted to the energy-related metabolism pathway, i.e., TCA cycle, and it is also possible to infer more enzymatic alterations in other metabolism pathways according to the abundance ratios of metabolic reactant pairs provided by MSI-based *in situ* metabolomics. The ratiometric MSI approach may provide a high throughput way to decipher the complicated metabolic reprogramming in ischemia at both metabolic and enzymatic levels.

## Data Availability

The original contributions presented in the study are included in the article/Supplementary Material, further inquiries can be directed to the corresponding author.
