# Supplementary material for: Ratiometric Mass Spectrometry Imaging for Stain-Free Delineation of Ischemic Tissue and Spatial Profiling of Ischemia-Related Molecular Signatures
Source: Front Chem. 2021 Dec 21;9:807868. doi: 10.3389/fchem.2021.807868 (PMC8724055; doi:10.3389/fchem.2021.807868)
Supplement: Supplementary file 1 [file Presentation1.pdf]

Supplementary Material

1 Contents

Supplementary Table 1 Statistical results and MS/MS fragment ions of metabolites in TCA cycle

Supplementary Figures 1-8 The MS/MS spectra of metabolites in TCA cycle.

Supplementary Figure 9 Extracted ion chronograms of malate ion at *m/z* 133.014, fumarate ion at *m/z* 115.0034 and their ratio, respectively, within 1.6-min scan time of MCAO rat brain section using AFADESI-MSI.

Supplementary Figure 10 OPLS-DA score plots and permutation results based on positive ion mode and negative ion mode AFADESI-MSI data of ischemic and healthy MCAO brain.

Supplementary Figure 11 Wide coverage metabolite analysis of ischemic tissues using AFADESI-MSI.

Supplementary Figure 12 Pathway analysis of significantly changed metabolite pathways after ischemia.

Supplementary Figure 13 HE staining of ischemic and healthy tissue.

Supplementary Figure 14 Distributions of NAA in ischemic and healthy heart, kidney and liver tissues by AFADESI-MSI.

MATLAB code

（1）Reconstruction of reactant pair

（2）Pixel segmentation for ratiometric MSI image

Supplementary Table 1 Statistical results and MS/MS fragment ions of metabolites in TCA cycle

Name	*m/z*	Formular	Adduct	FC	p value	MS/MS ions
malate	133.014	$C_4H_6O_5$	$[M-H]^-$	1.28	1.67E-10	31.9901;41.0034;43.0189; 44.9982;59.0138;71.0140; 72.9932;87.0089;89.0246; 115.0040;133.0150
succinate	117.0191	$C_4H_6O_4$	$[M-H]^-$	1.21	0.011751	31.9903;43.0100;55.0188; 73.0272;99.0089;99.9258
fumarate	115.0034	$C_4H_4O_4$	$[M-H]^-$	0.65	1.55E-25	31.9903;41.0031;55. 0189;71.0140;97.9310
aconitate	173.0086	$C_6H_6O_6$	$[M-H]^-$	0.95	0.170212	31.9903;41.0396;85.0293; 111.0087;129.0193
citrate*	191.0193	$C_6H_8O_7$	$[M-H]^-$	1.26	2.46E-10	41.0032;55.0189;57.0346; 67.0190;85.0294;87.0086; 111.0086;129.0194
2-oxoglutarate	145.0139	$C_5H_6O_5$	$[M-H]^-$	1.30	1.43E-06	41.0031;55.0191;57.0347; 71.0143;83.0141;87.0089; 101.0243
aspartate	132.0302	$C_4H_7NO_4$	$[M-H]^-$	0.74	7.83E-12	71.0138;88.0401;114.0197 ;115.0035
N-acetyl aspartate	174.0408	$C_6H_9NO_5$	$[M-H]^-$	0.38	2.43E-77	58.0300;59.0140;88.0403; 114.0196;115.0036;130.05 10;156.0303

*Representing fragment ion of isocitrate (*m/z* 155.1000) corresponding to the neutral loss of two water molecules [1] did not show in MS/MS spectrum, therefore the ion of *m/z* 191.0193 is identified as citrate.

[1]. Bylund, D., S. H. Norstrom, S. A. Essen, U. S. Lundstrom (2007). Analysis of low molecular mass organic acids in natural waters by ion exclusion chromatography tandem mass spectrometry. *J. Chromatogr. A* 1176(1-2), 89-93.doi: 10.1016/j.chroma.2007.10.064.

Supplementary Figures 1-8 show MS/MS spectra of the putative metabolite ions in the TCA cycle using in-situ AFADESI-MS/MS and LC-MS/MS of the tissue homogenate, respectively. In most cases, the AFADESI-MS/MS can be used for identification of the metabolites by the appearance of their characteristic product ions in the mass spectra although sometimes there may be some interferent ions with very close *m/z* to the selected precursor ions (less than 0.4 Da) that make the spectra look more complicated. We could not get MS/MS spectrum of metabolite ion with very low intensity, like *m/z* 145.01 (about 3E3) in the tissue section, therefore LC-MS/MS of these ions in the tissue homogenate is applied as a supplementary.

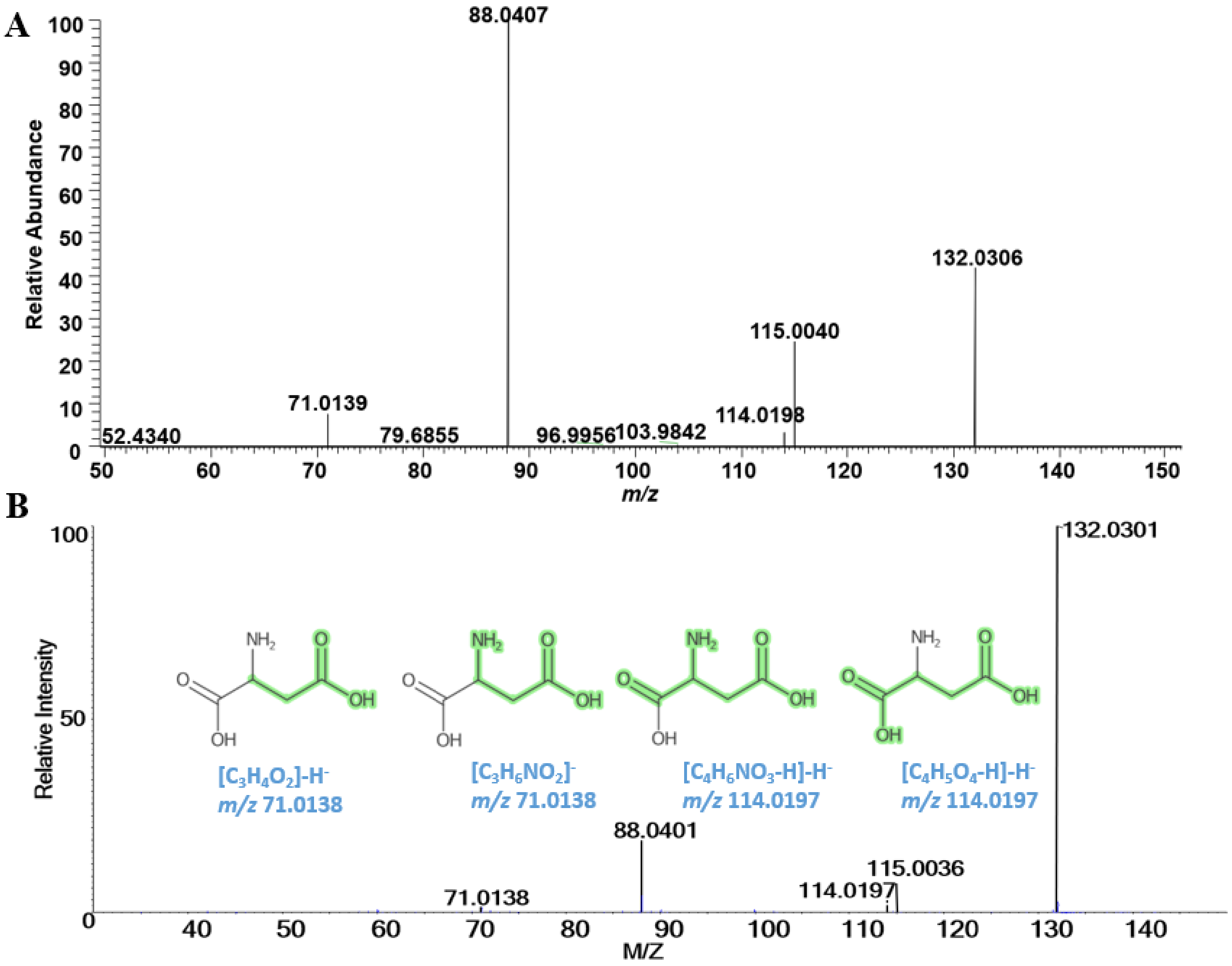

Supplementary Figure 1 Negative-ion mode MS/MS spectra of metabolite ions at *m/z* 132.03, annotated as aspartate by (A) in-situ AFADESI-MS/MS of the tissue section, and (B) LC-MS/MS of the tissue homogenate, respectively. Signals of fragment ions as marked were matched with the MetFrag (an in silico fragmentation for computer assisted identification of metabolite mass spectra, https://msbi.ipb-halle.de/MetFrag/) with human metabolome database.

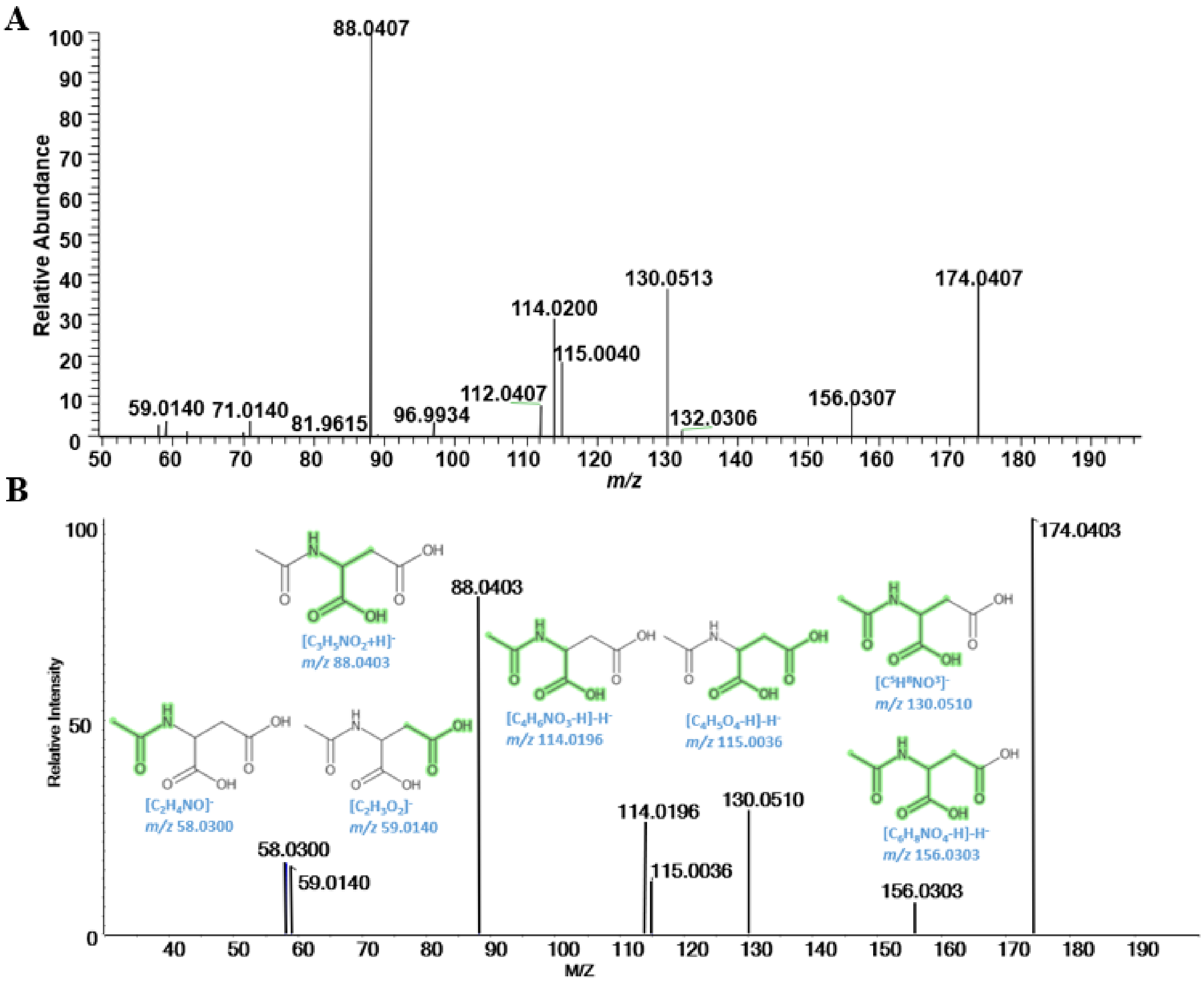

Supplementary Figure 2 Negative-ion mode MS/MS spectra of metabolite ions at *m/z* 174.04, annotated as N-acetyl aspartate by (A) in-situ AFADESI-MS/MS of the tissue section, and (B) LC-MS/MS of the tissue homogenate, respectively. Signals of fragment ions as marked were matched with the MetFrag (an in silico fragmentation for computer assisted identification of metabolite mass spectra, https://msbi.ipb-halle.de/MetFrag/) with human metabolome database.

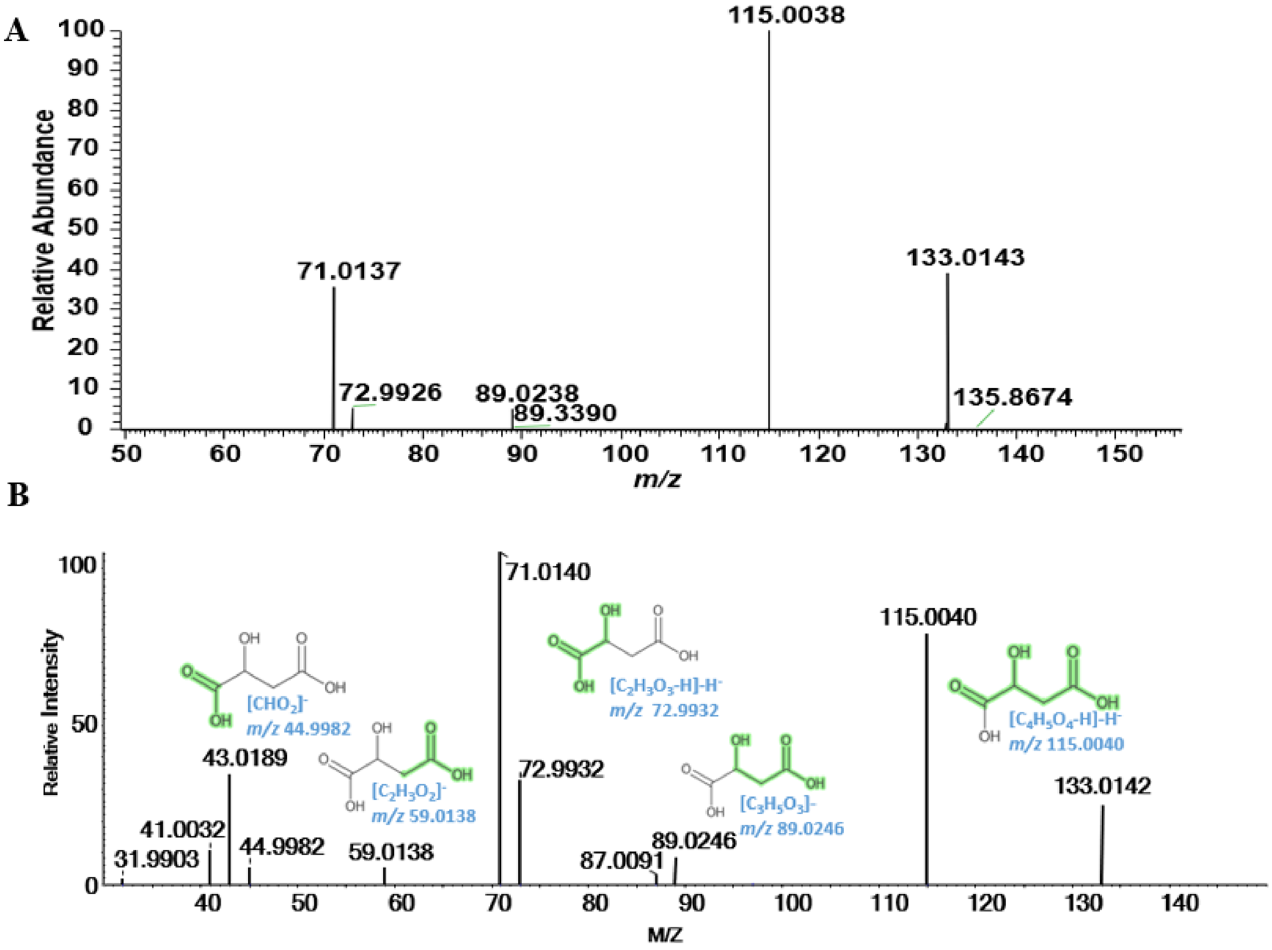

Supplementary Figure 3 Negative-ion mode MS/MS spectra of metabolite ions at *m/z* 133.01, annotated as malate by (A) in-situ AFADESI-MS/MS of the tissue section, and (B) LC-MS/MS of the tissue homogenate, respectively. Signals of fragment ions as marked were matched with the MetFrag (an in silico fragmentation for computer assisted identification of metabolite mass spectra, https://msbi.ipb-halle.de/MetFrag/) with human metabolome database.

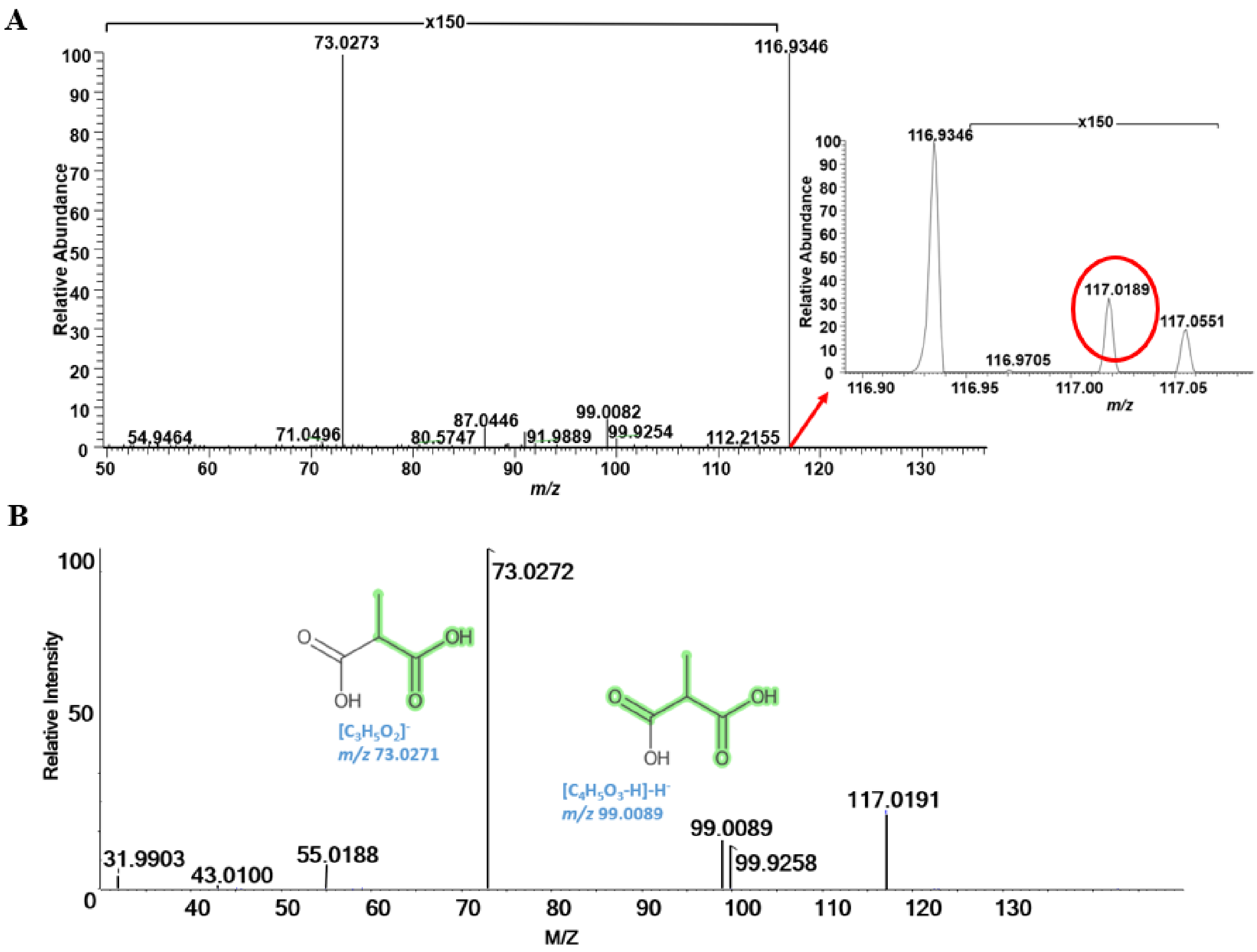

Supplementary Figure 4 Negative-ion mode MS/MS spectra of metabolite ions at *m*/*z* 117.02, annotated as succinate by (A) in-situ AFADESI-MS/MS of the tissue section, and (B) LC-MS/MS of the tissue homogenate, respectively. Signals of fragment ions as marked were matched with the MetFrag (an in silico fragmentation for computer assisted identification of metabolite mass spectra, https://msbi.ipb-halle.de/MetFrag/) with human metabolome database.

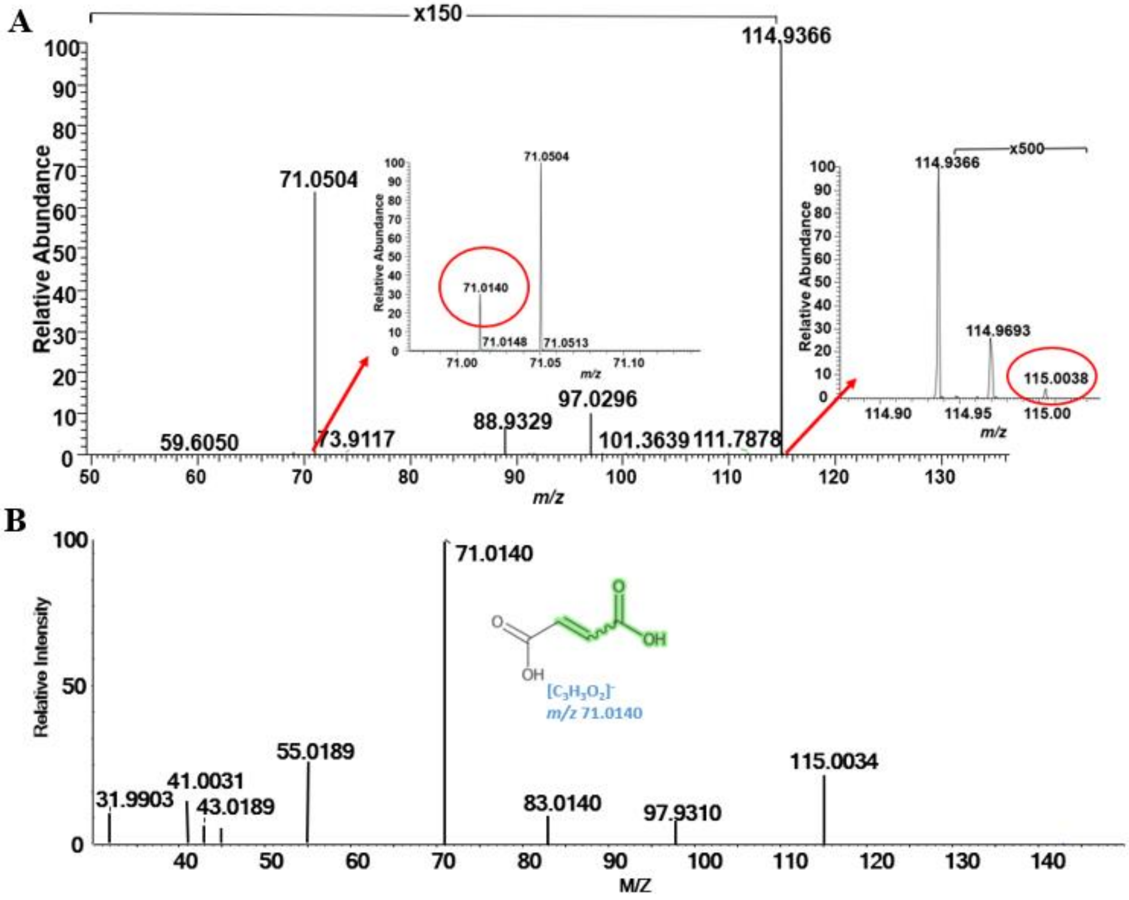

Supplementary Figure 5 Negative-ion mode MS/MS spectra of metabolite ions at *m*/*z* 115.00, annotated as fumarate by (A) in-situ AFADESI-MS/MS of the tissue section, and (B) LC-MS/MS of the tissue homogenate, respectively. Signals of fragment ions as marked were matched with the MetFrag (an in silico fragmentation for computer assisted identification of metabolite mass spectra, https://msbi.ipb-halle.de/MetFrag/) with human metabolome database.

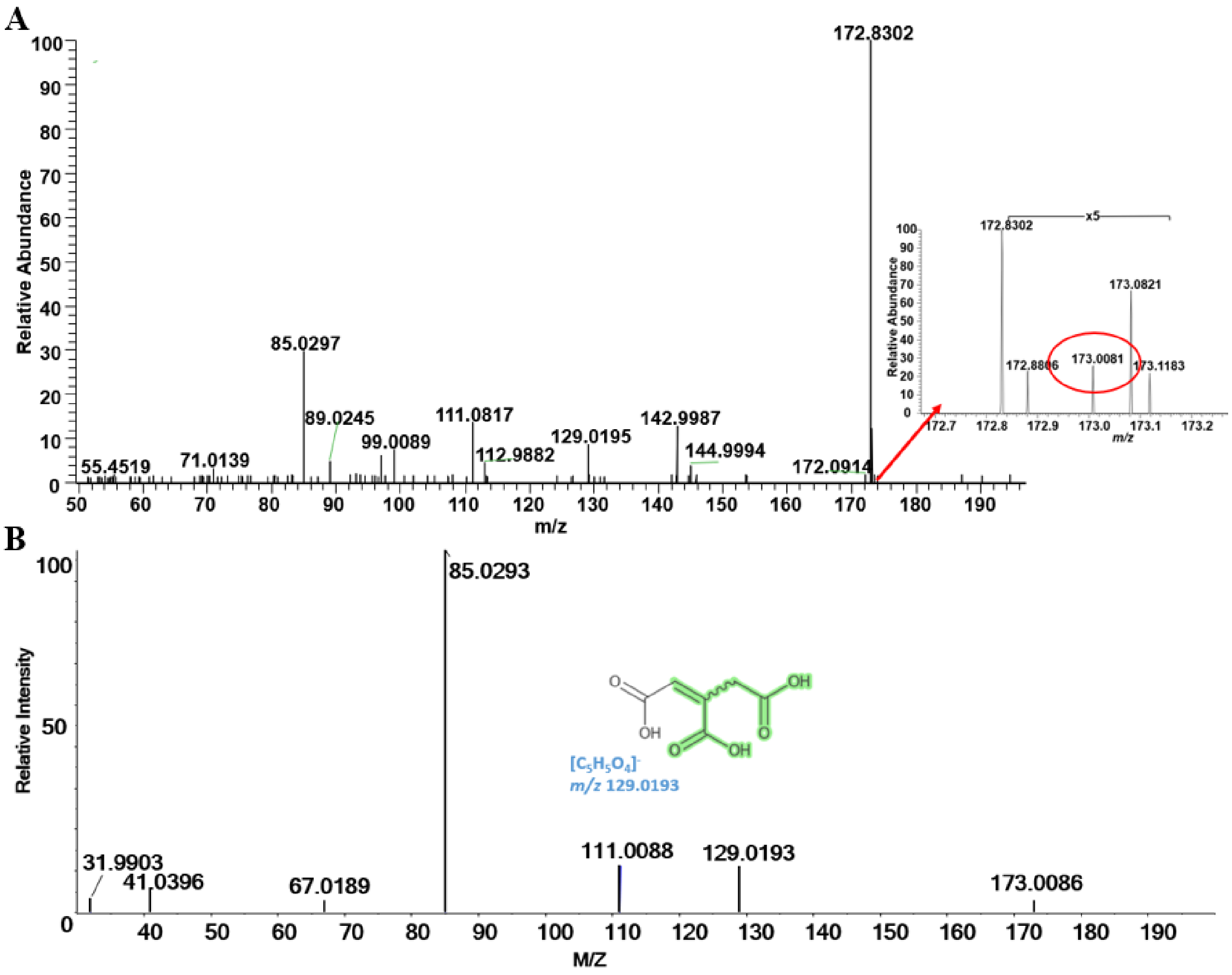

Supplementary Figure 6 Negative-ion mode MS/MS spectra of metabolite ions at *m/z* 173.01, annotated as aconitate by (A) in-situ AFADESI-MS/MS of the tissue section, and (B) LC-MS/MS of the tissue homogenate, respectively. Signals of fragment ions as marked were matched with the MetFrag (an in silico fragmentation for computer assisted identification of metabolite mass spectra, https://msbi.ipb-halle.de/MetFrag/) with human metabolome database.

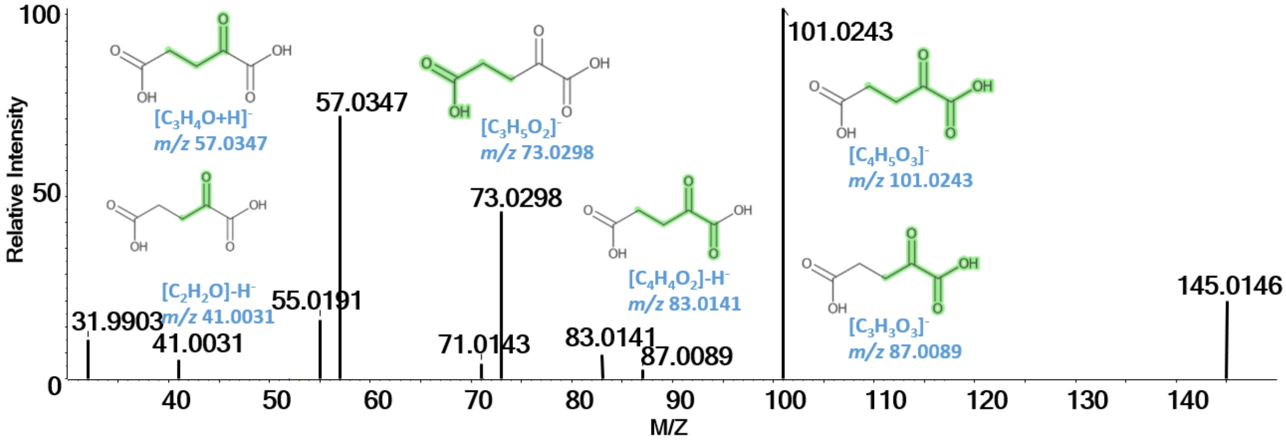

Supplementary Figure 7 Negative-ion mode MS/MS spectra of metabolite ions at *m/z* 145.01, annotated as 2-oxoglutarate by LC-MS/MS of the tissue homogenate. Signals of fragment ions as marked were matched with the MetFrag (an in silico fragmentation for computer assisted identification of metabolite mass s spectra, https://msbi.ipb-halle.de/MetFrag/) with human metabolome database. We did not get the in-situ AFADESI-MS/MS spectrum of metabolite ion at m/z 145.01 due to the quite low abundance of the precursor ion (about 3E3).

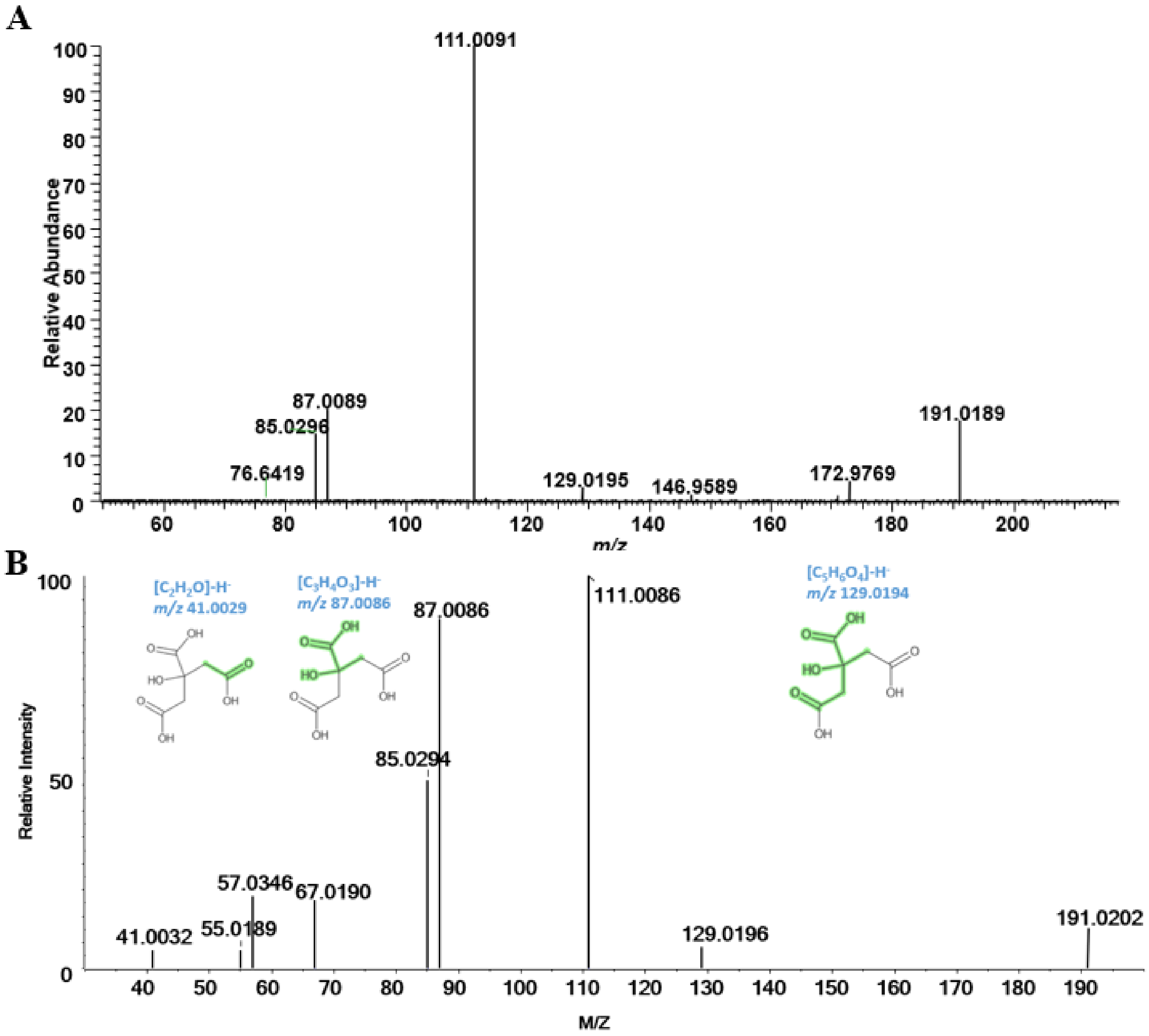

Supplementary Figure 5 Negative-ion mode MS/MS spectra of metabolite ions at *m/z* 191.02, annotated as citrate by (A) in-situ AFADESI-MS/MS of the tissue section, and (B) LC-MS/MS of the tissue homogenate, respectively. Signals of fragment ions as marked were matched with the MetFrag (an in silico fragmentation for computer assisted identification of metabolite mass spectra, https://msbi.ipb-halle.de/MetFrag/) with human metabolome database.

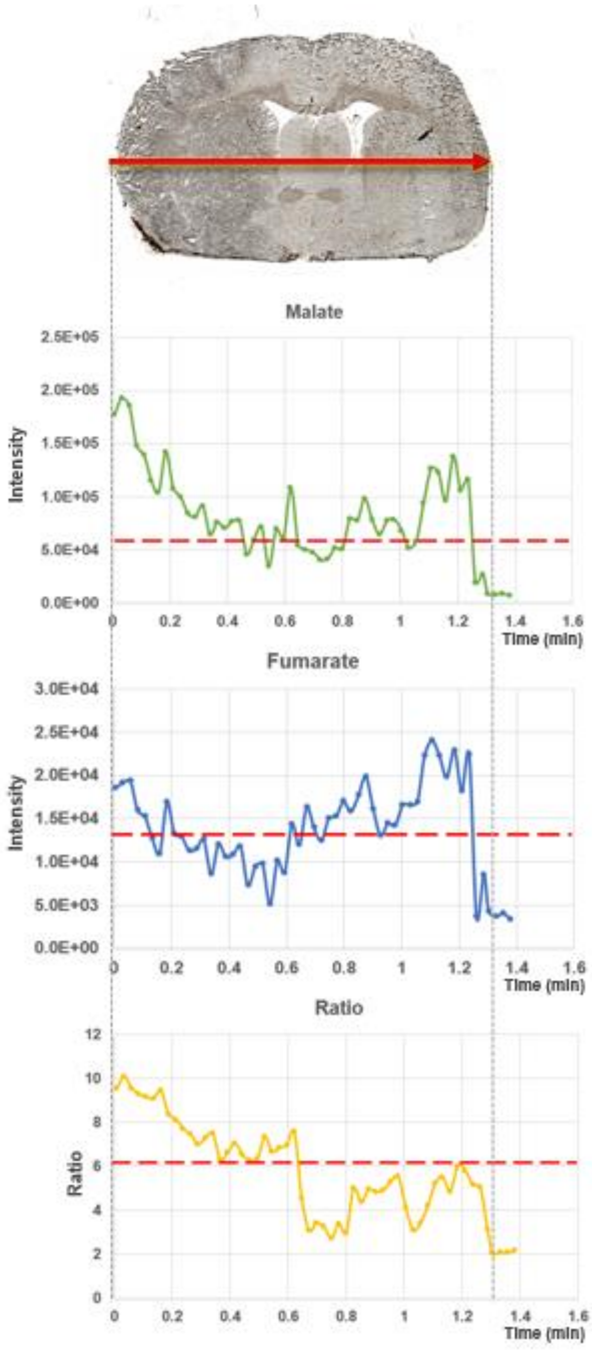

Supplementary Figure 9 Extracted ion chronograms of malate ion at *m*/*z* 133.014, fumarate ion at *m*/*z* 115.0034 and their ratio, respectively, within 1.6-min scan time of MCAO rat brain section using AFADESI-MSI. Red dotted line for cut-off value.

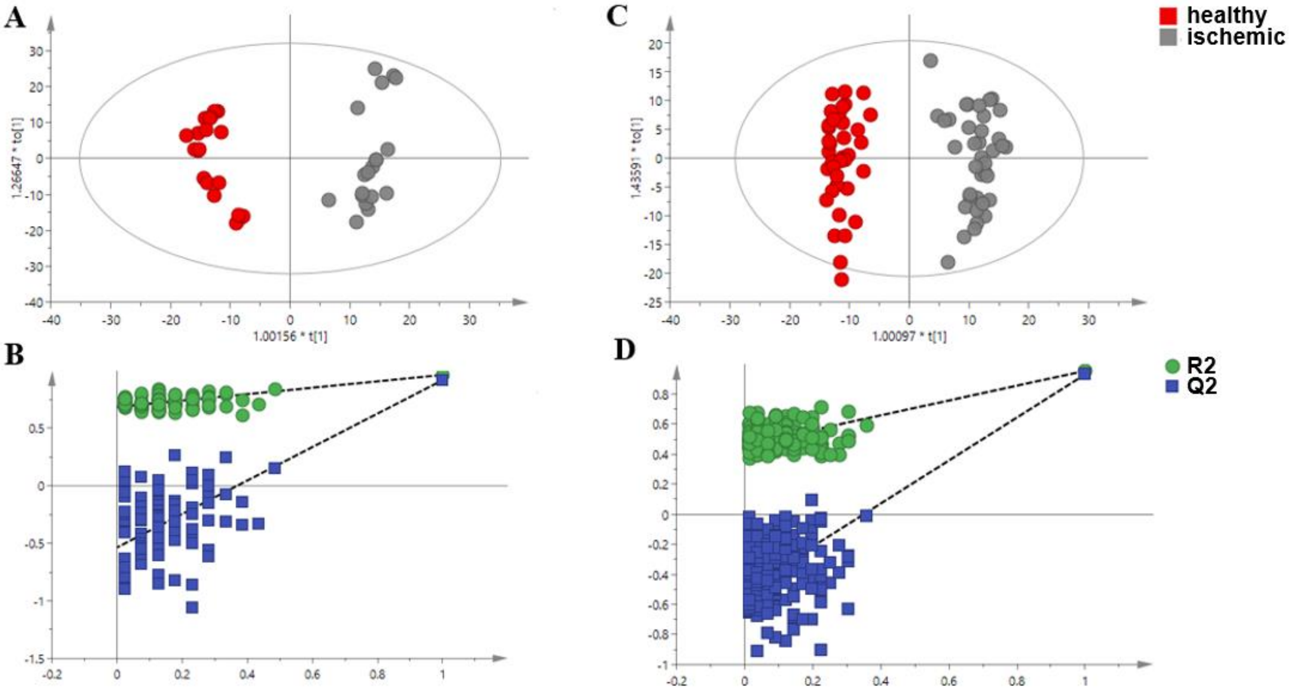

Supplementary Figure 10 OPLS-DA score plots and permutation results (with 200 times permutation) based on (A, B) positive-ion mode and (C, D) negative-ion mode AFADESI-MSI data of MCAO brains.

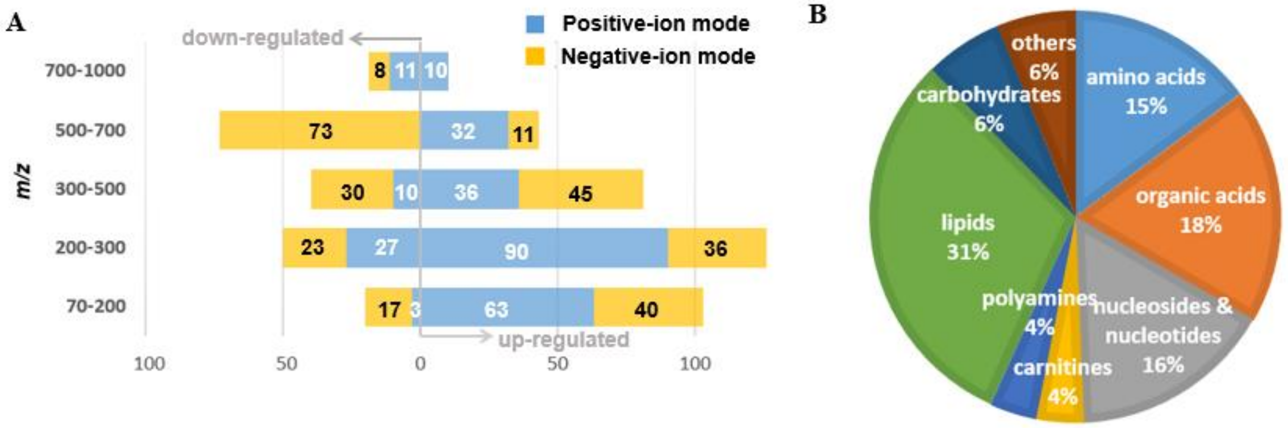

Supplementary Figure 11 Wide coverage metabolite analysis of ischemic tissues using AFADESI-MSI. (A) Statistical histogram of significantly altered ion features in ischemic area compared to healthy tissues in positive- and negative-ion mode AFADESI-MSI analysis. A total of 565 ion features were found significantly changed after ischemia. (B) Pie chart represents the types of annotated metabolite and their proportions in MCAO rat brain tissues.

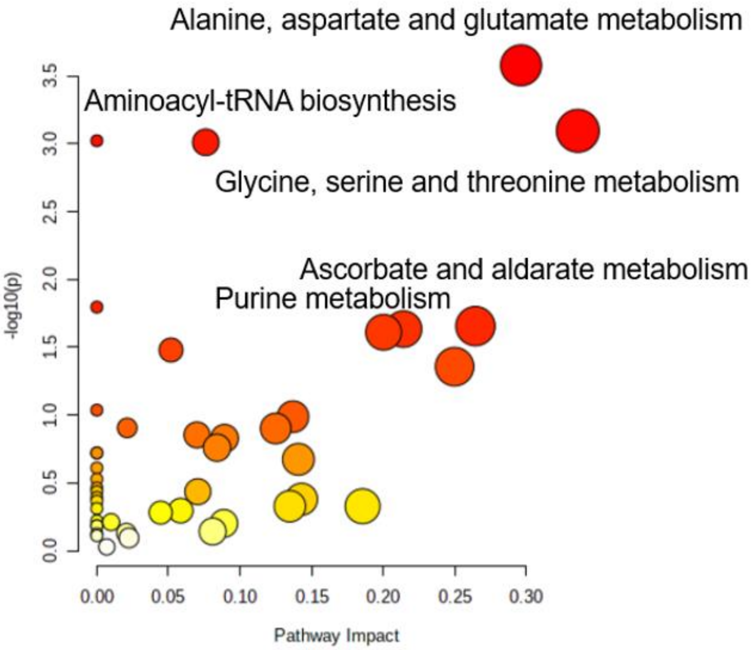

Supplementary Figure 12 Pathway analysis of significantly changed metabolic pathways after ischemia. The circle color is based on the metabolite p value and the circle size is based on its pathway impact value.

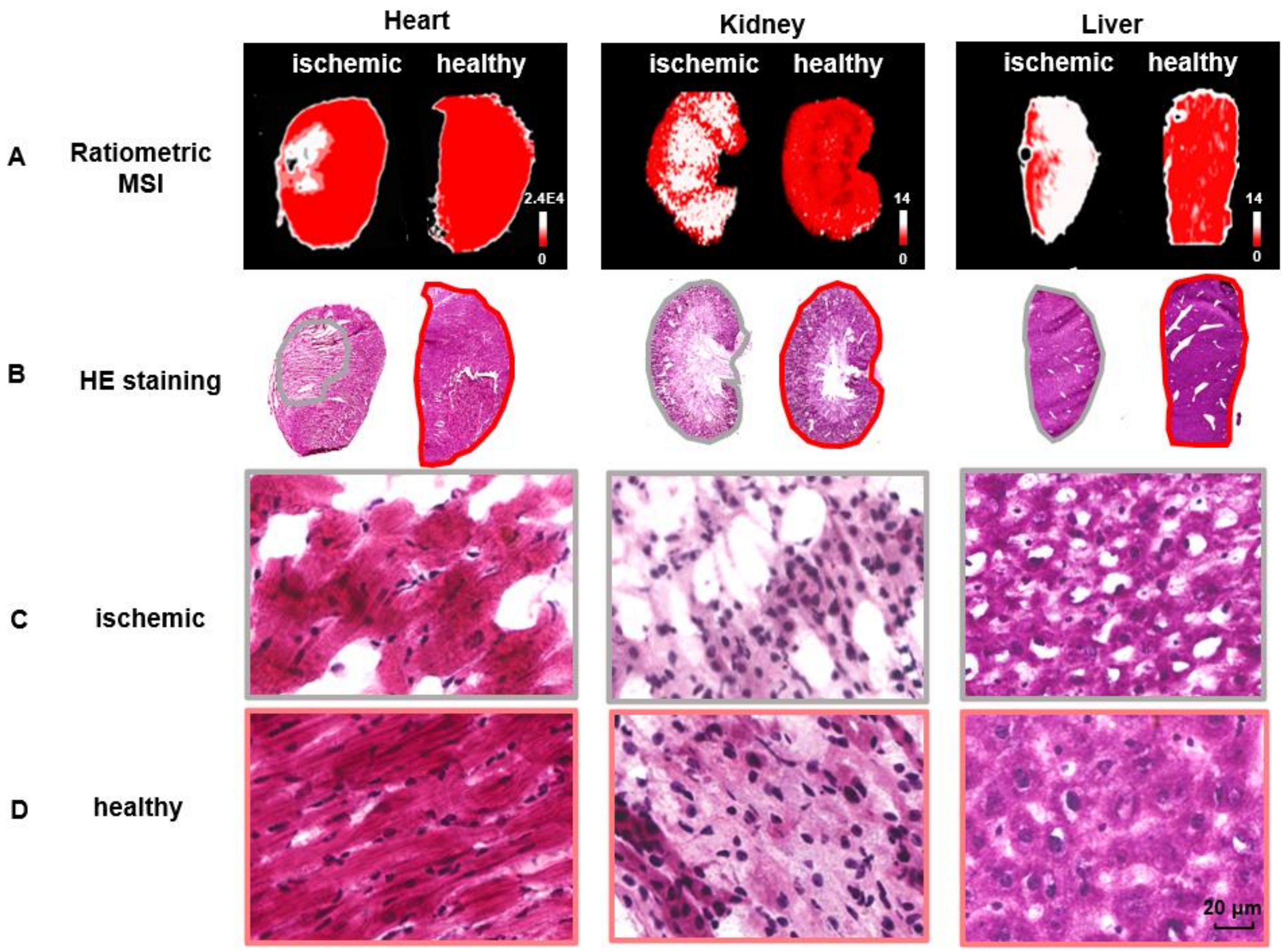

Supplementary Figure 13 Ratiometric MS images for ischemic delineation and adjacent HE staining of ischemic and healthy regions in rat myocardial ischemia and reperfusion model, mouse renal ischemia and reperfusion model, rat liver warm ischemia model, and their sham groups.

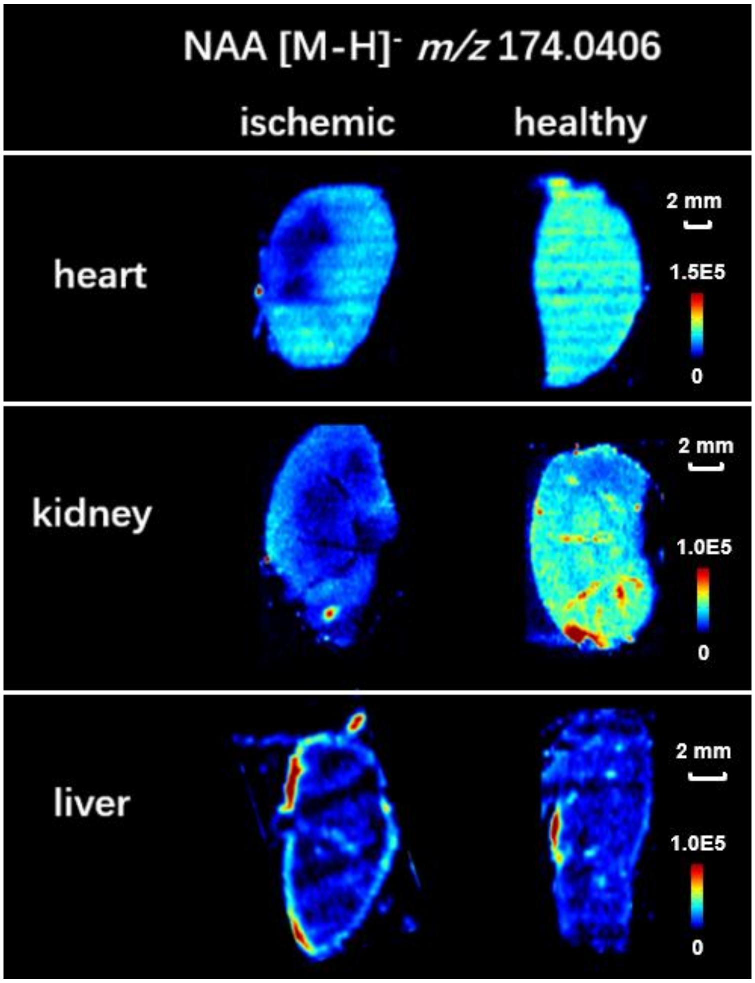

Supplementary Figure 14 Distributions of NAA in ischemic and healthy heart, kidney and liver tissues by AFADESI-MSI.

MATLAB code

（1）Reconstruction of reactant pair

```
substrate=[] % 2D data set of substrate
product=[]% 2D data set of product
ratio=product./substrate %pixel by pixel ratio of reactant pair
r2=interp2(ratio)
imagesc(r2)
```

（2）Pixel segmentation for ratiometric MSI image

```
imrgb=zeros(x,y,3); % x-pixel number in raw, y- pixel number in column
for i=1:x
for j=1:y
if a(i,j)<0
imrgb(i,j,1)=0;
imrgb(i,j,2)=0;
imrgb(i,j,3)=0;% set background to black
else if a(i,j)>cut-off value
imrgb(i,j,3)=255;
imrgb(i,j,1)=255;
imrgb(i,j,2)=255; %set ischemic tissue to white
else
imrgb(i,j,1)=255;
imrgb(i,j,2)=0;
imrgb(i,j,3)=0; % set normal tissue to red
   end
   end
```

```
    end
    end
figure(1);imshow(imrgb)
```
